# Microsatellite based molecular epidemiology of *Leishmania infantum* from re-emerging foci of visceral leishmaniasis in Armenia and pilot risk assessment by ecological niche modeling

**DOI:** 10.1371/journal.pntd.0009288

**Published:** 2021-04-19

**Authors:** Katrin Kuhls, Olga Moskalenko, Anna Sukiasyan, Dezdemonia Manukyan, Gayane Melik-Andreasyan, Liana Atshemyan, Hripsime Apresyan, Margarita Strelkova, Anja Jaeschke, Ralf Wieland, Marcus Frohme, Sofia Cortes, Ara Keshishyan

**Affiliations:** 1 Division of Molecular Biotechnology and Functional Genomics, Technical University of Applied Sciences Wildau, Wildau, Germany; 2 Research Platform Data Analysis & Simulation, Leibniz Centre for Agricultural Landscape Research (ZALF), Müncheberg, Germany; 3 Research Institute of Epidemiology, Virology and Medical Parasitology after A.B. Alexanyan, Ministry of Health, Yerevan, Armenia; 4 Eurasia International University, Yerevan, Armenia; 5 National Center of Disease Control and Prevention, Ministry of Health,Yerevan, Armenia; 6 Yerevan State Medical University after Mkitar Herats, Yerevan, Armenia; 7 Martsinovsky Institute of Medical Parasitology, Tropical and Vector-borne Diseases, Sechenov First Moscow State Medical University, Moscow, Russia; 8 Department of Biogeography, University of Bayreuth, Bayreuth, Germany; 9 Global Health & Tropical Medicine, Instituto de Higiene e Medicina Tropical, Universidade Nova de Lisboa, Lisbon, Portugal; Universiteit Antwerpen, BELGIUM

## Abstract

**Background:**

Visceral leishmaniasis (VL) is re-emerging in Armenia since 1999 with 167 cases recorded until 2019. The objectives of this study were (i) to determine for the first time the genetic diversity and population structure of the causative agent of VL in Armenia; (ii) to compare these genotypes with those from most endemic regions worldwide; (iii) to monitor the diversity of vectors in Armenia; (iv) to predict the distribution of the vectors and VL in time and space by ecological niche modeling.

**Methodology/Principal findings:**

Human samples from different parts of Armenia previously identified by ITS-1-RFLP as *L*. *infantum* were studied by Multilocus Microsatellite Typing (MLMT). These data were combined with previously typed *L*. *infantum* strains from the main global endemic regions for population structure analysis. Within the 23 Armenian *L*. *infantum* strains 22 different genotypes were identified. The combined analysis revealed that all strains belong to the worldwide predominating MON1-population, however most closely related to a subpopulation from Southeastern Europe, Maghreb, Middle East and Central Asia. The three observed Armenian clusters grouped within this subpopulation with strains from Greece/Turkey, and from Central Asia, respectively. Ecological niche modeling based on VL cases and collected proven vectors (*P*. *balcanicus*, *P*. *kandelakii)* identified Yerevan and districts Lori, Tavush, Syunik, Armavir, Ararat bordering Georgia, Turkey, Iran and Azerbaijan as most suitable for the vectors and with the highest risk for VL transmission. Due to climate change the suitable habitat for VL transmission will expand in future all over Armenia.

**Conclusions:**

Genetic diversity and population structure of the causative agent of VL in Armenia were addressed for the first time. Further genotyping studies should be performed with samples from infected humans, animals and sand flies from all active foci including the neighboring countries to understand transmission cycles, re-emergence, spread, and epidemiology of VL in Armenia and the entire Transcaucasus enabling epidemiological monitoring.

## Author summary

Leishmaniasis is a vector-borne disease caused by protozoan parasites of the genus *Leishmania*. In Armenia visceral leishmaniasis (VL) is re-emerging since 1999 after a long break of 30 years, with 167 cases recorded until 2019. Molecular diagnosis of VL was implemented only in 2016, and the causative agent was identified as *L*. *infantum*. In the present study we expanded the investigation of the causative agent to a characterization at strain level and the identification of its phylogenetic position among the *L*. *infantum* genotypes circulating worldwide. This is the first study addressing genetic diversity and population structure of *L*. *infantum* in Armenia and in Transcaucasia. Armenia is an extremely interesting region due to its bio-geographic specificities e.g. the high number of different climates in this small mountainous country and the observed high diversity of sand fly species, part of which occurring in very high altitudes. Ecological niche modeling based on registered VL cases and sand fly vectors collected in active VL foci revealed that the risk of further spread of VL is very high due to climate change. Studies of this region should be expanded to enable targeted control measures.

## Introduction

Leishmaniasis, a vector-borne disease caused by obligatory intracellular protozoan parasites of the genus *Leishmania*, is endemic in about 98 countries of the World [1]. The disease has different clinical manifestations, depending mainly on the species of the pathogen, ranging between visceral leishmaniasis (VL), the most severe form which is mostly fatal if untreated, the mostly self-healing cutaneous leishmaniasis (CL) characterized by localized lesions of the skin and the muco-cutaneous leishmaniasis (MCL) which affects mucous membranes.

In Armenia anthroponotic CL (ACL) was officially registered for the first time in 1920 in the Shirak district [2], and a total of 135 cases were recorded between 1938 and 1970, mainly in the cities Kapan and Goris (district Syunik) [3–5]. Zoonotic CL (ZCL) caused by *L*. *major* has never been recorded neither in Armenia, nor in whole Transcaucasia. VL was firstly reported in 1913 [6], and between 1935 and 1969 919 cases were registered in 62 villages or cities in 16 of the former 37 districts of the country, with Yerevan being the most active focus with 81% of all cases [7]. Infected dogs were identified as the main reservoir, and foxes, jackals and wolves as wild animal reservoirs [8–10]. There are about 18 species of sand flies in Armenia occurring in different frequencies as reported by different authors (**S1 Table**) [8,11–16]. Of these only *P*. *kandelakii* and *P*. *balcanicus* were considered as the most likely vectors for VL in the Caucasus region based on their wide distribution, the presence in places with VL cases, and their ability to feed on humans and dogs [8,17–19]. Only recently, in 2012, molecular evidence of *P*. *balcanicus* and *P*. *kandelakii* infected by *L*. *infantum* was provided for specimens collected in neighbouring Georgia [20]. Apart from these two species, also *P*. *tobbi*, *P*. *neglectus*, *P*. *transcaucasicus* and *P*. *perfilievi* (all subgenus *Larroussius)*, as well as *P*. *brevis and P*. *simici* (both subgenus *Adlerius)* were considered to transmit VL in the Southern Caucasus [21,22].

Between the mid-1950s and the 1960s a series of wide-scale control measures were conducted against CL and VL including treatment of patients, insecticidal treatment and trapping of sick dogs in parallel with massive spraying campaigns against malaria vectors. These actions resulted in an almost complete elimination of CL in Transcaucasia, and since 1963 no CL cases were detected in Armenia. Also VL morbidity was significantly reduced, as between 1969 and 1999 no case was reported in the country [23–26]. Recently, two detailed historical overviews were published about leishmaniasis in Armenia and Transcaucasia including all relevant references, of which most are in Russian [21,26].

After a break of 30 years VL re-emerged in Armenia in 1999, with a case reported in the Lori district [26,27]. Until 2016, 116 indigenous and 99 imported VL cases were recorded mainly in the districts Lori, Tavush, and Syunik [26,27]. The majority of cases (97%) were children up to 10 years old. In all the active foci seroprevalence studies revealed considerably high numbers of infected dogs [28]. In the Republic Nagorno-Karabakh 82 VL cases were reported in 2004–2013 [29]. The current trend of VL re-emergence is also being observed in the other Transcaucasian countries as Georgia and Azerbaijan [1,21,30]. Apart from the recognized and reported case numbers, underreporting is suspected especially in Armenia and Azerbaijan. Cutaneous leishmaniasis has not been registered in Armenia any more after 1963, apart from two imported cases from Syria and the Republic Nagorno-Karabakh.

Until 2015 diagnosis of VL in Armenia was based exclusively on the clinical picture and microscopic examination of Giemsa-stained smears from bone marrow aspirates. Only from 2015 on serological tests (ELISA, rK39) were continuously implemented. PCR-based methods were applied for diagnosis in 2016 for the first time in Armenia [26,31,32]. Moreover, for a long time it was only assumed based on clinical and epidemiological data that the causative agent of VL in Armenia was *L*. *infantum* [8,23]. This could be finally proven by using PCR-RFLP of the Internal Transcribed Spacer 1 (ITS1) region of the ribosomal DNA and by sequencing [26].

The objective of the present study was to get more insights into the genetic diversity and the population structure of *L*. *infantum* in Armenia and to compare the Armenian genotypes with those from endemic regions all over the World. The knowledge about the genotypes circulating in Armenia will enable conclusions about the mode of spread and the origin of the strains for further epidemiological monitoring. Discrimination of genotypes at strain level is possible only by using highly variable markers. One of the most powerful and discriminative DNA-based methods for strain differentiation and population genetics is the analysis of the highly variable, co-dominant microsatellite markers [33,34]. Multilocus Microsatellite Typing (MLMT) has been successfully applied to differentiate *L*. *infantum* populations in the Mediterranean region of Europe and North Africa, the Middle East, Central Asia, the Far East and South America [35–43]. In the present study we have applied MLMT for the very first time for the Transcaucasian region.

In addition we present the results of vector surveys in active VL foci of Armenia. According to previous entomological monitoring an increase in vector populations of *P*. *balcanicus*, *P*. *kandelakii* and *P*. *papatasi* was observed, especially in Yerevan, the Ararat valley, and the districts Syunik and Lori [44]. Based on the occurrence data of vectors and VL cases, and considering climate conditions and future climate trends, we inferred ecological niche models to predict the spatiotemporal spread of the vectors in the present and in the future, to enable risk assessment for VL in Armenia.

## Material and methods

### Ethics statement

This study was subject to ethical review by the Ethical Committee of the Yerevan State Medical University after Mkhitar Heratsi (Approval Nr. 9, 01.07.2016). In all cases *Leishmania* was isolated from patients as part of normal diagnosis and treatment. The aims of the study were explained to the responsible person of each minor and informed consent was obtained in written form. All samples have been anonymised, laboratory codes have been used for the samples along with the corresponding demographic patient data.

### Human samples and study area

All suspected cases are reported to and all diagnostic examinations are carried out in the Research Institute of Epidemiology, Virology and Medical Parasitology named after A.B. Alexanyan, Ministry of Health, Yerevan. In 2017 this institute was affiliated to the Reference Laboratory Center of the National Center for Disease Control and Prevention in Yerevan. Between 2012 and 2016 all clinically suspected VL patients were hospitalized in the Infectious Clinical Hospital “Nork” in Yerevan. In total 91 cases were confirmed by microscopy of Giemsa-stained slides of bone marrow aspirates in this time period [26]. Confirmed cases were treated with meglumine antimoniate (Glucantime).

In 2015/16 molecular diagnosis based on PCR-RFLP of the Internal Transcribed Spacer 1 (ITS1) of the ribosomal DNA was applied for the first time in Armenia for 25 of these clinically suspected cases [26]. Twenty two of them were positive by microscopy of the bone marrow aspirates showing different parasitic loads, and by ITS1-PCR with the detection of *Leishmania* DNA. Further Restriction Fragment Length Analysis (RFLP) and selective sequencing of the ITS1 PCR products identified the parasites as *L*. *infantum* [26].

**Fig 1:**
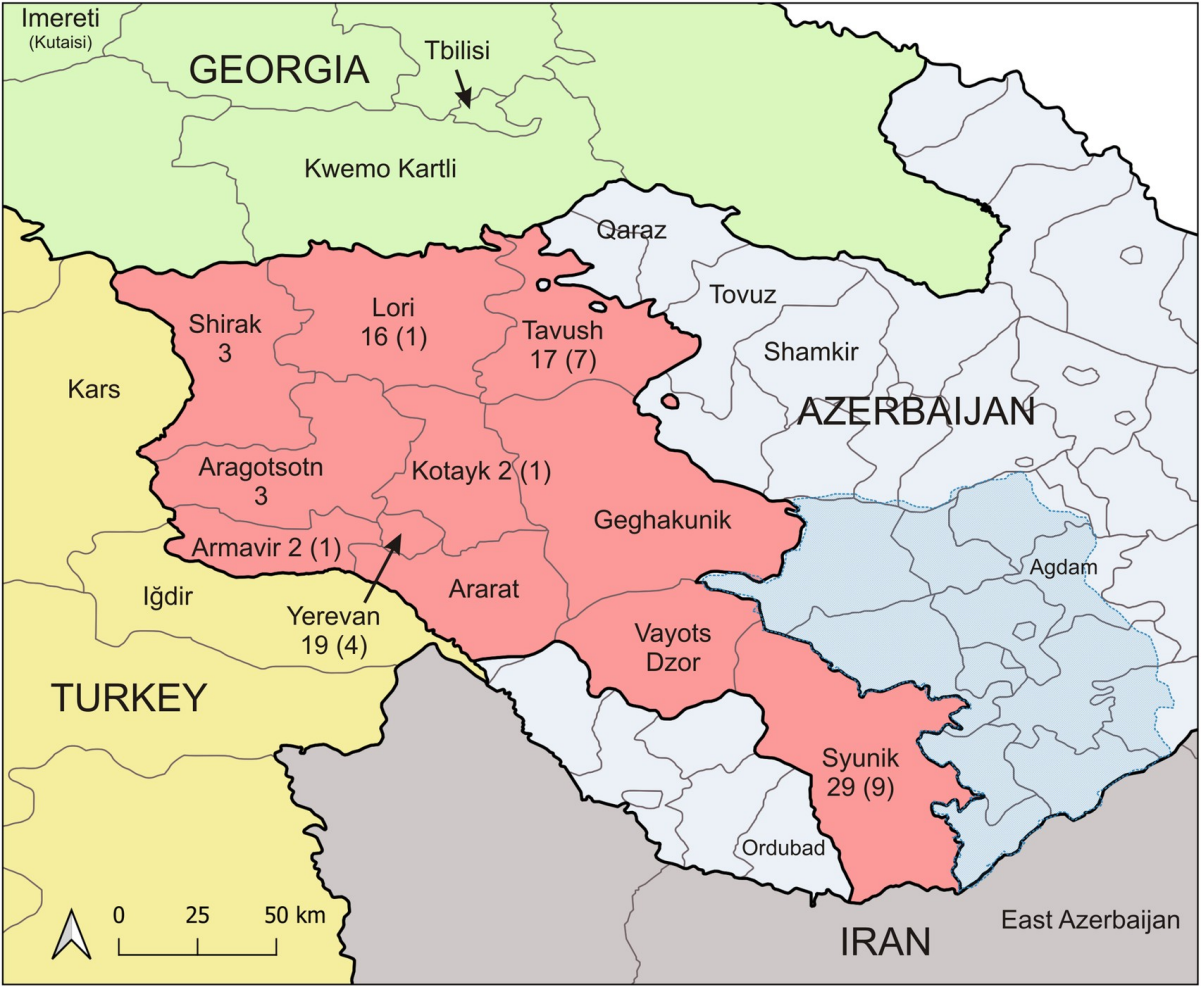
Geographical origin of the studied samples of visceral leishmaniasis. Map showing the distribution of the 91 registered cases of visceral leishmaniasis in 2009–2016 among the districts of Armenia. These cases were used for ecological niche modeling. Out of these 91 cases, 23 were analyzed by Multilocus Microsatellite Typing (in parentheses). Adjacent foci of visceral leishmaniasis in the neighboring countries are indicated. The map was generated by the use of shapefiles available from https://gadm.org/download_country_v3.html.

These 22 samples and three additional, previously not characterized ones, were included in the present study for further multilocus microsatellite typing (MLMT) (**Table 1** and **Fig 1)**. The patients came from different regions of Armenia—Syunik, Lori, Tavush, Yerevan, Armavir, and Kotayk and were mostly children under 3 years old. The *L*. *infantum* strain MHOM/ES/1993/PM1 was used as reference in the MLMT experiments.

**Table 1:** Designation, patient characteristics, geographic origin and population assignment by MLMT of the studied Armenian *L*. *infantum* samples.

Patients labcode	Year of collection	Age [years]	Gender	District	Locality	Population assignment (*K* = 2) **
Arm3	2013	9 months	female	Syunik	Kapan	1
Arm5	2014	3.2	female	Syunik	Karahunj	1
Arm6	2012	2	female	Syunik	Kapan	1
Arm7	2013	0.9	male	Syunik	Kapan	2a
Arm8	2012	12	male	Lori	Akhtala	1
Arm9	2012	1.8	female	Yerevan	Nоr Nork district	1
Arm10	2014	1.7	male	Tavush	Baganis	2a
Arm11	2014	1	female	Tavush	Baganis	1
Arm12	2015	3.3	female	Armavir	Armavir	2a
Arm13	2015	2.5	male	Syunik	Kapan	1
Arm14	2015	5	female	Syunik	Kapan	1
Arm15	2015	1.5	male	Yerevan	Chorenaci str.	failed*
Arm16	2015	2	female	Yerevan	Chachatryan str.	1
Arm17	2015	2	male	Yerevan	Kanaker district	1
Arm18	2015	2	male	Yerevan	Tcarav Aghbyur str.	1
Arm19	2015	2	female	Kotayk	Abovyan	2b
Arm20	2015	2.8	male	Syunik	Goris	2b
Arm21	2014	2.8	female	Syunik	Goris	2b
Arm22	2013	2	male	Syunik	Goris	2b
Arm23	2016	3	female	Tavush	Ijevan	1
Arm24	2016	1	male	Tavush	Koti	1
Arm25	2016	1	male	Tavush	Koti	failed*
Arm26	2016	3	male	Tavush	Koghb	2a
Arm27	2016	3.9	female	Tavush	Sarigyugh	2a
Arm28	2016	2.8	male	Tavush	Berd	2a

* MLMT failed because of insufficient amount of DNA isolated from the clinical material

** ARM-pop1 and ARM-pop2 both were further tested for substructures. 2a and 2b are the two subpopulations inferred for population ARM-pop2. ARM-pop1 was not further divided into subpopulations.

### PCR amplification assays and fragment analysis of the microsatellite markers

DNA was isolated from stained slides with smears of bone marrow aspirates as described elsewhere [26,45,46] with an additional purification step using a DNA purification kit (Qiagen) to avoid inhibition. The DNA was stored at -20°C until use.

The standard set of 14 markers (Lm2TG, TubCA, Lm4TA, Li41-56, Li46-67, Li22-35, Li23-41, Li45-24, Li71-33, Li71-5/2, Li71-7, CS20, kLIST7031, LIST7039) specific for the *Leishmania donovani* complex was used for amplification of microsatellite containing fragments, as previously described [35,36]. PCRs were performed with fluorescence-conjugated forward primers. Screening of length variations of the amplified markers was done by automated fragment analysis using an ABI3130XL sequencer and the ABI PRISM GeneMapper software (Applied Biosystems, Foster City, CA). The repeat numbers calculated for the 14 loci were assembled into a multilocus microsatellite profile for each sample under study.

### Microsatellite data analysis

Population structure was inferred using a Bayesian model-based clustering approach implemented in the bioinformatic programme STRUCTURE [47]. This algorithm identifies genetically distinct populations on the basis of allele frequencies. Genetic clusters are constructed from the genotypes identified, estimating for each strain the fraction of its genotype that belongs to each cluster. The probability estimates in this study were based on 200,000 Markov Chain Monte Carlo iterations with a “burn-in” period of 20,000 iterations. For each *K* ten iterations were performed and the most appropriate number of populations was determined by calculation of Δ*K*, which is based on the rate of change in the log probability of data between successive *K* values [48].

Phylogenetic analysis was based on microsatellite genetic distances, calculated with the program POPULATIONS 1.2.28 (http://bioinformatics.org/~tryphon/populations) for the number of repeats within each locus using the Chord-distance [49], which follows the infinite allele model (IAM). Neighbor-joining trees (NJ) were constructed with the POPULATIONS software and visualized with MEGA4 [50]. Microsatellite markers as well as populations were analysed with respect to diversity of alleles (*A*), expected and observed heterozygosity (*H*_e_ and *H*_o_) applying GDA (http://hydrodictyon.eeb.uconn.edu/people/plewis/software.php). Genetic differentiation and gene flow was assessed by *F*-statistics [51,52] with the corresponding *p-*values using the MSA software [53].

All analyses were performed using three different data sets: 1. Armenian VL samples separately **(Table 1)**; 2. 537 strains including *L*. *infantum* strains from endemic regions all over the Old World, the 23 Armenian samples and 12 representatives of *L*. *donovani* from the Middle East, Southeastern Europe and East Asia; 3. 176 strains representing the populations which are most closely related to the Armenian samples (subset of 2). The data of the 514 strains were available from our MLMT database created during former own studies [36–43,54,55], supplemented with the MLMT profiles of *L*. *infantum* from China published by other authors [56]. It also included two strains from Iran, AKH-1-inf from Ardabil and AKH-3-inf from Meshkin shahr (both in the Ardabil province) which were typed in this study (kindly provided by M. Akhoundi, Dept. Parasitology, Hôpital Avicenne–Hôpitaux Universitaires Paris-Seine-Saint-Denis, France). In total 22 countries were represented **(Table 2)**. The *L*. *infantum* strains from the New World (Brazil, Venezuela, Paraguay, Colombia, Costa Rica, Panama, Honduras and Belize) were not included, since it was shown previously, that they are genotypically related to the Western European strains from Portugal, Spain, Italy and France, which are part of the present dataset [40]. A few *L*. *donovani* strains were added as outgroup to the dataset. *Leishmania donovani* and *L*. *infantum* both are belonging to the *L*. *donovani* species complex. The included *L*. *donovani* strains are from the Middle East, Cyprus and from China and are phylogenetically more closely related to *L*. *infantum* non-MON-1 strains than to the main *L*. *donovani* populations from East Africa and the Indian subcontinent [54,55].

**Table 2:** Overview of the 537 strains included in the global MLMT analysis and assignment to the three main populations inferred by STRUCTURE.

Region	Country	*L*. *infantum*	*L*. *donovani*	STRUCTURE *K* = 3		
				pop1-537 non-MON-1	pop2-537 MON-1	pop3-537 MON-1
Western Europe	Spain	66		18	48	
	Portugal	60		4	56	
	France	32		3	29	
	Italy	30		2	25	3
	Malta	1		1		
Southeastern Europe	Greece	39			36	3
	Cyprus	22	*6*	*6*		22
	Albania	3			3	
Eastern Europe	Crimea		*1*	*1*		
**Transcaucasia**	**Armenia**	**23**			**3**	**20**
Middle East	Turkey	23		7	14	2
	Israel/Palestine	48	*1*	*1*	2	46
	Iran	2				2
	Iraq		*2*	*2*		
	Saudi Arabia		*1*	*1*		
North Africa	Algeria	55		20	5	30
	Tunisia	36		15	1	20*
	Morocco	33		1	2	30
	Egypt	1			1	
Central Asia	Uzbekistan	20				20
	Tajikistan	2				2
East Asia	China	29	*1*	21**+*1*	8	
**total**		***525***	***12***			

* including seven strains from Tunisia with shared membership in pop1-537 and pop3-537

** including 5 strains from China with shared membership in pop1-537 and pop3-537; *L*. *donovani* strains in italics, all strains included in the subgroup-analysis (176 strains) are underlined

### Vector studies

Between 2009 and 2015 field studies were performed in order to monitor the occurrence of sand fly species and specifically of the potential and primary vectors of *L*. *infantum* in Armenia. The studies were performed in active VL foci of six districts: Lori, Kotayk, Shirak, Ararat, Yerevan, and Syunik and carried out according to epidemiological indications. Sand flies were collected during the main season of activity in Armenia between May and October using sticky traps (castor oil traps) installed outdoors and indoors of resting places. The specimens were removed from the sticky traps using a brush dipped in 96% ethanol, washed with 96% ethanol to remove any remaining castor oil and stored in 75% ethanol at 4°C until mounting them on slides. Species identification was performed by microscopy based on morphology and morphometric analysis mainly of the male genitalia and the female spermathecea using the identification keys by Perfiliev and Artemiev & Neronov [22,57].

### Spatiotemporal species distribution models and risk assessment

Species distribution modeling was performed including all 91 microscopically confirmed VL cases from 2009–2016 [26] and 100 records of vectors of *L*. *infantum* (*P*. *balcanicus*- 64; *P*. *kandelakii*– 36) collected during field studies in active VL foci in 2009–2015 **(S2 and S3 Tables)** as occurrence data. The geographical location (latitude, longitude, altitude) was obtained using GeoLocator (http://tools.freeside.sk/geolocator/). The positive occurrence points were often concentrated on a single geographical coordinate, because the exact position of the acquisition of the infection could not be determined. In order to achieve a spatial resolution of such positive points, a grid of 8 cells with grid sizes of 1km*1km is laid over the area (D8 area) and the locations of disease cases in the D8 environment were distributed randomly, i.e. 1 km to the north, 1 km to the northeast, 1 km to the east etc. We considered a height difference < 150 m, with the exception of the four large cities Yerevan, Kapan, Goris, and Alaverdi, where the difference was between 160 and 280 m. This ensured that only climatically similar areas were included in the modeling. Additional points were not introduced into the modeling. This approach only ensured that the existing records were less clustered. In the case of the sand flies, the amount of added positive points was calibrated according to the amount of caught specimens, to consider the abundance in the respective sites. Three classes have been used: 2–4, 5–8, 9–13 specimens and one, two or three points have been added, respectively.

Based on a literature review of ecological and environmental factors affecting sand fly survival, development, behaviour and activity, and further analysis of the impact of twelve preselected variables specifically for Armenia by hierarchical partitioning, the following seven of the total number of 19 bioclimatic variables of WorldClim (http://www.worldclim.org/bioclim) [58] were selected for the current and future projections: BIO1—Annual Mean Temperature, BIO5 –Maximum Temperature of Warmest Month, BIO6 –Minimum Temperature of Coldest Month, BIO10—Mean Temperature of Warmest Quarter, BIO11—Mean Temperature of Coldest Quarter, BIO12—Annual Precipitation, BIO14 –Precipitation of Driest Month, and BIO16 –Precipitation of Wettest Quarter. The highest impact among the variables had those related to temperature. All climate data have a spatial resolution of 30 arc-seconds (approximately 1 km^2^) and are based on WorldClim v1.4 (https://www.worldclim.org/data/v1.4/worldclim14.html) [58].

Four modeling algorithms were applied: GLM (Generalized Linear Model) [59], GBM (Generalized Boosted Model) [60], and RF (Random Forest) [61] included in the biomod2 R-Package version 3.3–7 (https://CRAN.R-project.org/package=biomod2) [62,63] and MAXENT (Maximum-Entropy-Modeling) [64]. Biomod2 adds a number of pseudo absence points to the data points. This enables a supervised training of the models. In the present analysis 434 randomly chosen pseudo absence points were added. Ensemble modeling was performed using the four algorithms with each algorithm having the same weighting (25%). This simple allocation of the weights has proven to be successful and simple heuristics often lead to better results than complex optimizations [65].

The available occurrence data were used to calculate the current and the future projections (2041–2060) for VL. Future data for the bioclimatic variables are CMIP5 (Coupled Model Intercomparison Project) downscaled future climate projections done with WorldClim v1.4 as baseline climate. For future projections, the Global Climate Model (GCM) mpi-esm-lr (http://ccafs-climate.org/data_spatial_downscaling/) and the Shared Socio-economic Pathway (SSP) emission scenario RCP 4.5 were used. RCP 4.5 is an intermediate scenario expecting a raise in the global mean surface temperature between 1.1°C and 2.6°C until the end of the century [66]. Mapping of the derived projections was done using the Quantum GIS software (QGIS 3.12.0-Bucureşti, http://www.qgis.org/de/site/).

## Results

### Multilocus Microsatellite Typing and population structure analyses

#### Genetic diversity of *L*. *infantum* in Armenia

**Fig 2:**
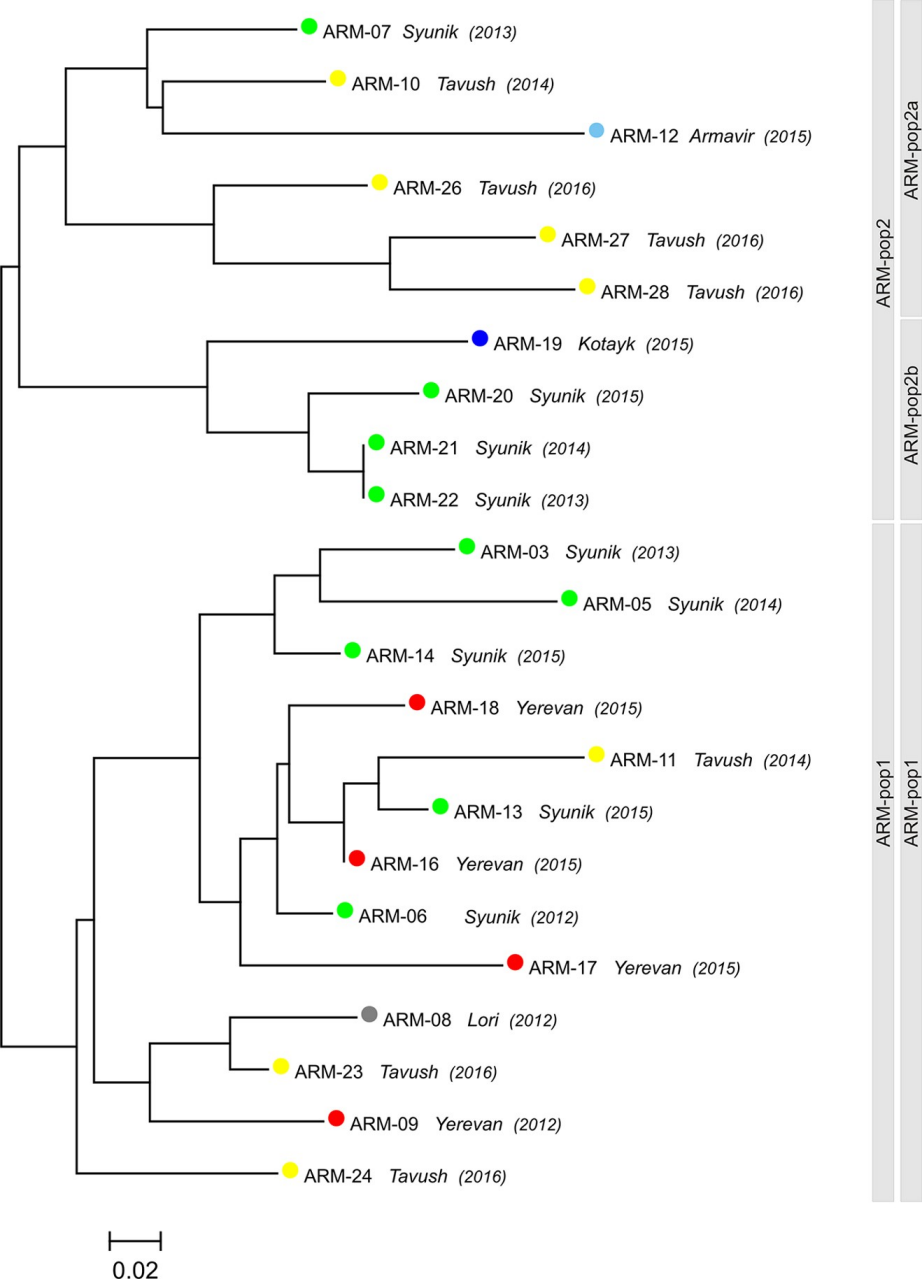
Diversity of genotypes of *Leishmania infantum* from clinical samples of 23 patients with visceral leishmaniasis. Samples are from active foci in six districts of Armenia (shown in Fig 1) marked by different colors. The year of occurrence of the infection is indicated in brackets. The neighbor joining tree is based on multilocus microsatellite typing with a standard set of 14 microsatellite markers specific for the *Leishmania donovani* complex. Populations as inferred by STRUCTURE are indicated by bars next to the tree. According to Δ*K* the most probable number of populations is two (*K =* 2, left bar). In addition also the result of the analysis for substructures is shown (right bar). ARM-pop2a and ARM-pop2b are the two subpopulations inferred for population ARM-pop2. ARM-pop1 was not further divided into subpopulations.

PCR products of all 14 microsatellite markers were obtained for 23 of the 25 included Armenian samples (**Tables 1 and S5**). Two samples (ARM-15, ARM-25) had to be excluded because of insufficient DNA amounts. Descriptive analysis of the MLMT data per microsatellite marker is shown in **Table 3**. The number of alleles per locus is varying from 1 to 4, with an average of 2.29. The most variable markers with four alleles are Lm2TG, Lm4TA, Li 41–56, Li 22–35, Li 23–41 and Li 45–24, which is in agreement with the observations in previous studies on *L*. *infantum* from other countries. The observed heterozygosity was very low (mean *H*_o_ = 0.031) and always much lower than the expected heterozygosity (mean *H*_e_ = 0.301). Only for three of the 14 markers *H*_o_ was higher than zero. This observed heterozygote deficiency most probably is due to population subdivision (Wahlund effect) or endogamy and should be tested in future with an increased number of samples from Armenia. Twenty two different genotypes (MLMT profiles) were identified within the 23 samples, which is a quite high genetic diversity considering the small territory of Armenia **(Fig 2**). Only two of the samples (ARM-21 and ARM-22) showed identical MLMT profiles. Both originated from the same city (Goris) in Syunik, however isolated in two successive years (2013 and 2014) from unrelated patients and distinct localities in the city. In the phylogenetic tree two main clusters could be observed, each of which showing further subclusters. There was neither a clear relationship between genotype and geographical origin of the samples, nor the year of isolation. Bayesian statistics revealed two main populations as inferred by Δ*K* calculation. Both were identical with the two main clusters observed in the neighbor joining tree, as indicated by the bars next to the tree in **Fig 2**. Only one of these two populations (ARM-pop2) was further subdivided into two groups (ARM-pop2a and ARM-pop2b) by substructure analysis. The new population ARM-pop2a included strains ARM-19, ARM-20, ARM-21 and ARM-22, three of which were from the city Goris in Syunik, isolated in three successive years, and the fourth from the city Abovyan in Kotayk. This group was also evident as subcluster of one of the two main clusters in the tree. The assignment of the respective strains for *K* = 2, and according to further substructure analysis is shown in **Table 1**.

**Table 3:** Descriptive analysis per locus of the 23 *L*. *infantum* samples from Armenia.

Marker	number of strains	repeat array	fragment sizes (alleles) [bp]	A	*H*_e_	*H*_o_
Lm2TG	23	TG 15,22,23,25	122, 136, 138, 142	4	0.509	0.087
TubCA	23	CA 9,13	80, 88	2	0.085	0
Lm4TG	23	TG 11,12,13,14	77, 79, 81, 83	4	0.639	0.261
Li 41–56 (B)	23	CA 10,11,12	90, 92, 94	3	0.669	0
Li 46–67 (C)	23	CA 9	80	1	0	0
Li 22–35 (E)	23	CA 12,15,16	92, 98, 100	3	0.371	0
Li 23–41 (F)	23	CA 13,14,17	79, 81, 87	3	0.541	0
Li 45–24 (G)	23	CA 15,16,20	105, 107, 115	3	0.532	0.087
Li 71–33 (P)	23	CA 10,11	103, 105	2	0.464	0
Li 71-5/1 (Q)	23	CA 9	110	1	0	0
Li 71–7 (R)	23	CA 12,13	98,100	2	0.162	0
CS20	23	CA 19	85	1	0	0
kLIST7031	23	CA 11	111	1	0	0
LIST7039	23	CA 14,15	205, 207	2	0.085	0
*mean*				*2*.*29*	*0*.*301*	*0*.*031*

A—number of alleles; *H*_o_—observed heterozygosity; *H*_e_—expected heterozygosity

#### Comparison of the Armenian *L*. *infantum* genotypes with genotypes from the main endemic regions of visceral leishmaniasis in the World

Bayesian analysis of the whole dataset (set 2) revealed three main populations (pop1-537, pop2-537, pop3-537) as inferred by Δ*K* calculation (**Table 2 and S1 Fig**). The first population (pop1-537) included all 12 *L*. *donovani* (from Iraq, Saudi Arabia, China, Crimea, Israel, Cyprus) and the non-MON-1 *L*. *infantum* strains from Turkey, Algeria, China, Tunisia, and Western Europe (Spain, Portugal, France, Italy and Malta). The second main population (pop2-537) comprised all MON-1 *L*. *infantum* from Spain, Portugal, France, and 27 of the 30 MON-1 strains from Italy. In addition it included the majority of strains from Southeastern Europe (36 of the 39 strains from Greece, all strains from Albania) and 14 of the 16 MON-1 strains from Turkey (of these 14 four showed shared alleles with population pop3-537). Three of the strains from Armenia (ARM-26, 27, 28) also were part of this population, however with shared membership with pop3-537. There were also single strains from Algeria (4), Tunisia (1), Morocco (2) and Israel/Palestine (2) in pop2-537. The third population (pop3-537) included MON-1 strains from Cyprus, Israel/Palestine, Algeria, Morocco, Tunisia, Iran, Egypt, Uzbekistan, Tajikistan and all remaining 20 strains from Armenia. It also included three of the strains from Greece, two of the strains from Turkey and three of the 30 strains from Italy (all from Sicily). Thirteen strains from China revealed shared memberships in all three populations with various proportions.

**Fig 3:**
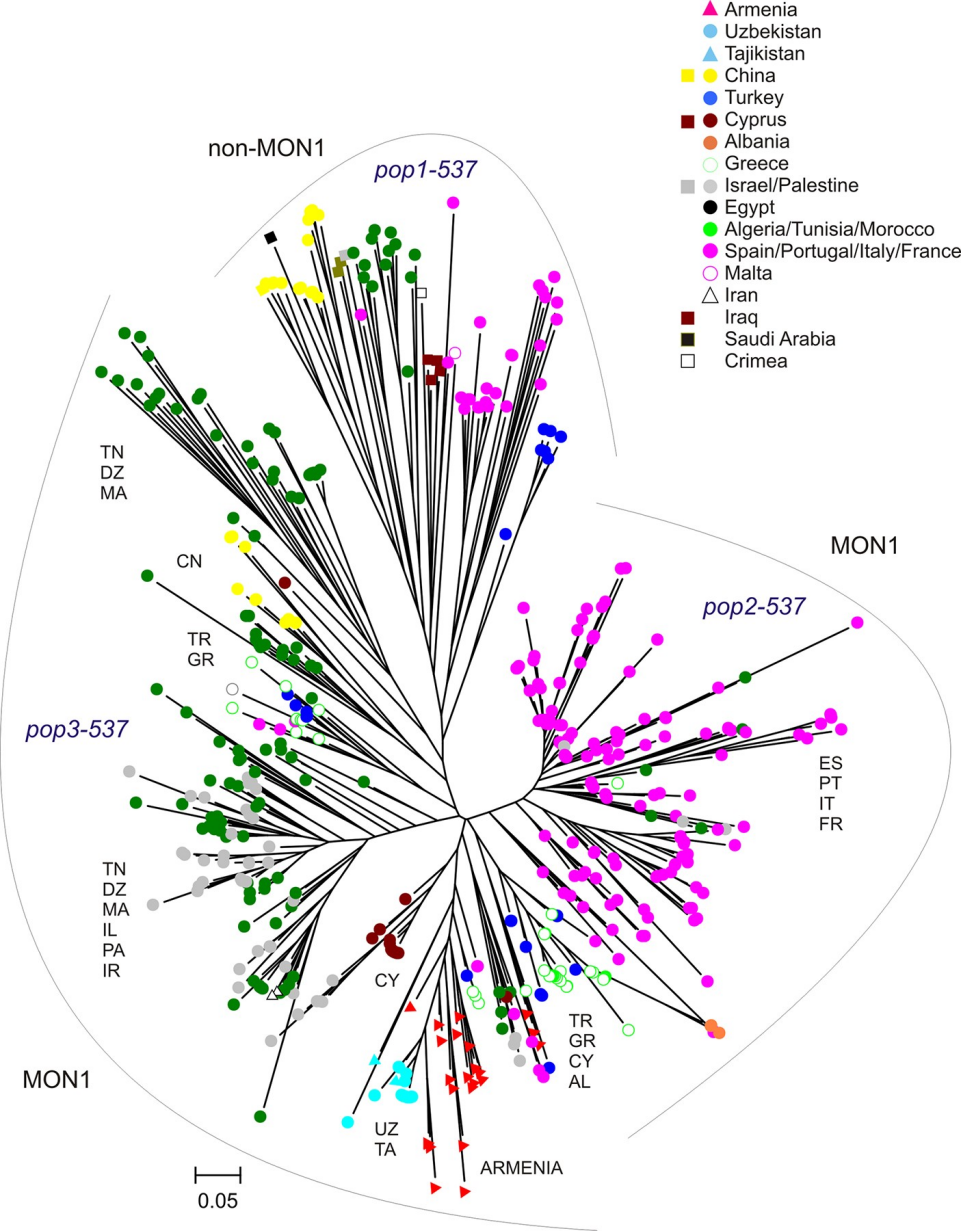
Neighbor joining tree inferred from MLMT profiles of 525 strains of *L*. *infantum* and 12 strains of *L*. *donovani* obtained with a standard set of 14 microsatellite markers specific for the *Leishmania donovani* complex. The tree shows the position of *L*. *infantum* from Armenia in comparison to the main endemic regions of visceral leishmaniasis in Europe, Africa and Asia. Strains typed by MLEE as *L*. *donovani* are marked by squares. The three populations inferred by STRUCTURE are indicated as pop1-537, pop2-537 and pop3-537 and marked by grey curves. UZ–Uzbekistan, TA–Tajikistan, CN–China, TR—Turkey, CY—Cyprus, AL—Albania, GR—Greece, IL—Israel, PA—Palestine, MA—Malta, DZ—Algeria, IR—Iran, ES—Spain, PT—Portugal, IT—Italy, FR–France.

The phylogenetic tree presented in **Fig 3** was inferred from the MLMT profiles of 537 strains. This tree shows that all *L*. *infantum* strains from Armenia are belonging to the MON-1 genotype, which is the dominating genotype of *L*. *infantum* all over the World. The Armenian strains including all three populations ARM-pop1, ARM-pop2a (except ARM-26, ARM-27, ARM-28) and ARM-pop2b are forming a separate cluster among the overall *L*. *infantum* population, including also all strains from Uzbekistan and Tajikistan. The group of Armenian genotypes can be clearly differentiated from the genotypes circulating in other countries. The branch leading to the Armenian and Uzbek/Tajik strains also included three strains from Greece, one strain from Turkey and two strains from Sicily. The tree also shows that the *L*. *infantum* strains from Armenia are part of a MON-1 subpopulation, which comprises strains from Turkey, Greece, Cyprus, the Balkans (along with ARM-26, ARM-27, ARM-28) and small subgroups of strains from Israel/Palestine, the Maghreb and Italy (Sicily). A more distant relationship exists to the cluster which includes the majority of strains from Israel/Palestine, the strain from Egypt, the two strains from Iran and a major part of the strains from Algeria/Tunisia/Morocco. Unfortunately, there are, apart from Turkey, any data available from the neighboring countries of Armenia, Georgia, and Azerbaijan. All strains of *L*. *donovani*, also those from geographically relatively adjacent countries or locations as Iraq or Crimea, are clustering within the non-MON-1 population and are not related to the Armenian strains. All strains from Western Europe (with few exceptions) are forming a well separated cluster in the tree which corresponds with pop2-537 from the Bayesian analysis. All *L*. *donovani* strains as well as all non-MON-1 *L*. *infantum* strains also are comprised in a distinct main cluster which corresponds with pop1-537.

#### Detailed analysis of the Armenian samples with its most closely related populations

A subset of 176 strains has been created in order to analyze the population structures in more detail and to elucidate the position of the three previously detected Armenian subpopulations (pop1, pop2a, pop2b). This subset included *L*. *infantum* strains from Turkey, Greece, Cyprus, Uzbekistan, Tajikistan, Albania, Israel/Palestine, Egypt, Iran and the 23 Armenian strains (**Table 2** –underlined numbers). These groups of strains were shown in **Fig 3** and in the Bayesian analysis (**Table 2 and S1 Fig**) to be most closely related to the Armenian samples. Strains from Greece and Turkey seem to hold an intermediate position between the Western European (pop2-537) and the North African (pop3-537) populations, because of varying assignment to these main populations in analyses of different datasets. Because of this and the geographic proximity, all Greek and Turkish strains were included. Strains from Algeria/Tunisia/Morocco were not used because they shared the main cluster with the strains from Israel/Palestine, which were included as representatives of this cluster.

**Fig 4:**
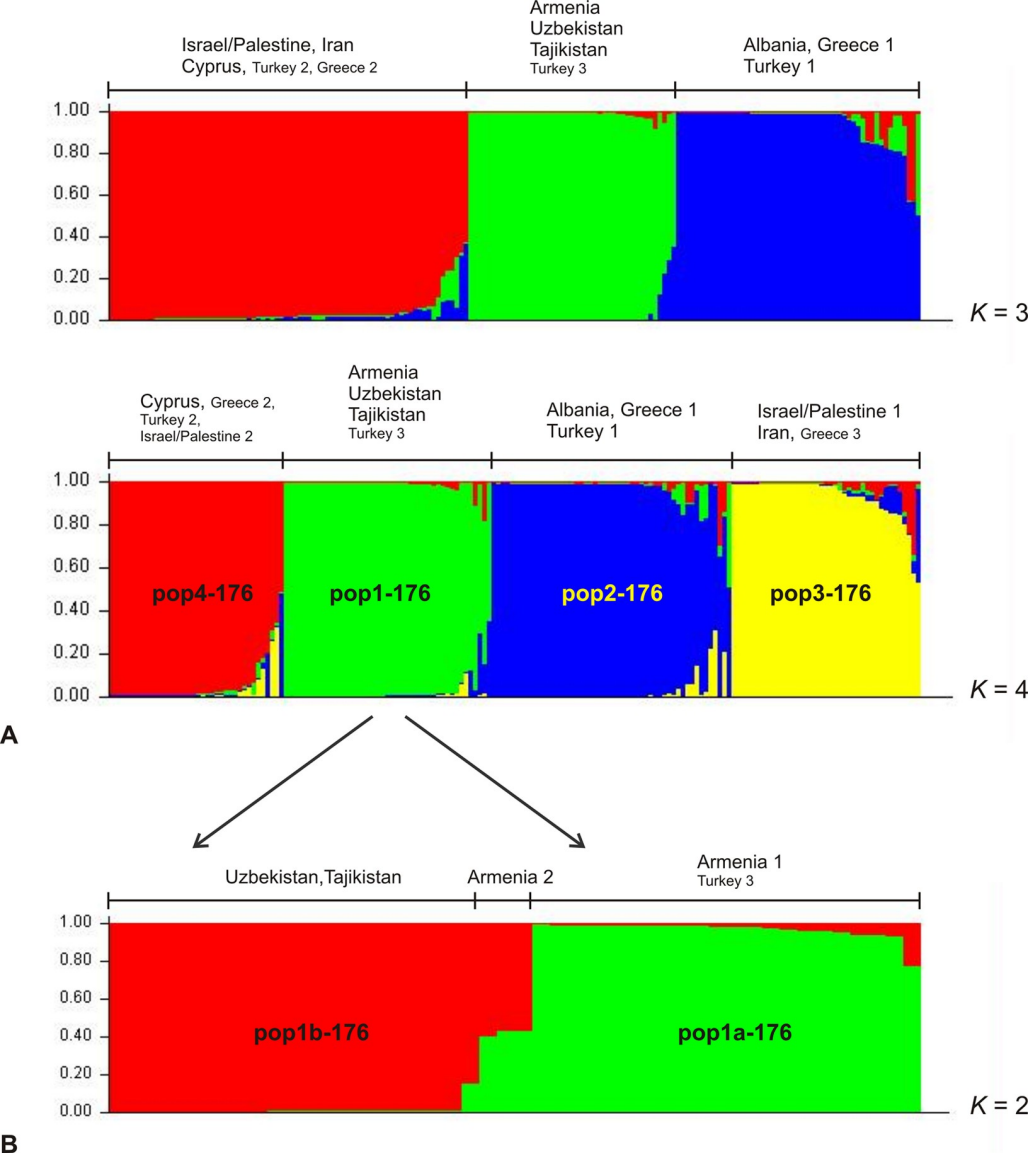
Estimated population structure of a subset of 176 *L*. *infantum* MON1 strains most closely related to the Armenian strains as inferred by Bayesian analysis of their MLMT profiles. In the barplots each strain is represented by a single vertical line divided into *K* colors, where *K* is the number of populations assumed. Each color represents one population. The length of the colors segment shows the strain’s estimated proportion of membership (Q) in that population. Strains are sorted by membership coefficient (Q). (A) According to Δ*K* the most probable number of populations is four (*K* = 4), in addition also *K* = 3 is shown. (B) Structure of subpopulation 1 (pop1-176) comprising the strains from Armenia, Uzbekistan, Tajikistan and Turkey3. According to Δ*K* the most probable number of populations is two (*K* = 2).

The first split in Bayesian inference (*K* = 2) identified the following two groups: (1) Greece (37 of 39 strains), Turkey (22 of 23), Armenia (all 23), Uzbekistan (all 20), Tajikistan (all 2), Egypt (the single strain); (2) Cyprus (all 22), Israel/Palestine (all 48), Iran (all 2), Greece (2 of 39), Turkey (1 of 23). At *K* = 3 the first of the above mentioned groups was further divided into two subgroups, first of which contained all strains from Uzbekistan, Tajikistan, Armenia and a single strain from Turkey (Turkey 3); and the second contained the strains from Greece (37 of 39 –Greece 1), Albania, Egypt and Turkey (21 of 23 –Turkey 1), whereas the second of the *K*2 groups remained unchanged (**Fig 4A**). Delta *K* calculation identified four distinct subpopulations based on allele frequencies **(Figs 4A and S2A)**. These four populations were also supported by *F*-statistics. All *F*_ST_-values were > 0.25 and significant (*p* = 0.0001) indicating strong genetic differentiation **(Table 4)**. All Armenian strains were found in the first of these four subpopulations (pop1-176), which also included all strains from Uzbekistan and Tajikistan and a single strain from Turkey (Turkey 3). The second population (pop2-176) comprised the majority of strains from Greece (33 of 39 –Greece 1), Turkey (21 of 23 –Turkey 1), all strains from Albania (3) and the single strain from Egypt. The third population (pop3-176) consisted of the majority of strains from Israel/Palestine (38 of 48 –Israel/Palestine 1), the two strains from Iran and a single strain from Greece (1 of 39 –Greece 3). All strains from Cyprus and few strains from Greece (5 of 39 –Greece 2), Turkey (1 of 23 –Turkey 2) and Israel/Palestine (10 of 48 –Israel/Palestine 2) formed the fourth population (pop4-176). Within pop1-176 strains ARM-25, ARM-26, ARM-27 (three of the six strains of ARM-pop2a) shared part of their alleles with the population pop2-176 (dominant population of Greece/Turkey) indicating a certain relationship to this group, as already observed in the analysis of the 537 strains.

Population pop1-176 was further tested for substructures. Two subpopulations were inferred by Δ*K* calculation (**Fig 4B and S2B**). Pop1a-176 comprised 20 of the 23 Armenian strains (ARM-pop1, ARM-pop2a), a single strain from Uzbekistan (with shared membership with a higher percentage in pop1a-176) and the single strains from Turkey (Turkey 3). Pop1b-176 included all strains from Uzbekistan, Tajikistan and three strains from Armenia (ARM-20, ARM-21, ARM-22 (ARM-pop2b)). The latter three strains had shared membership with pop1a-176, with a slightly higher percentage in pop1b-176. ARM-19 (ARM-pop2b) also showed shared membership, however with a higher percentage in pop1a-176.

**Table 4:** *F*_ST_ values and corresponding *p*-values for the four main populations inferred by STRUCTURE for the dataset of 176 strains.

*F*_ST_ -value	Pop1-176 (ARM)	Pop2-176	Pop3-176	Pop4-176
Pop1-176 (46)	0	0.478	0.507	0.428
Pop2-176 (51)	0.0001	0	0.543	0.431
Pop3-176 (41)	0.0001	0.0001	0	0.447
Pop4-176 (38)	0.0001	0.0001	0.0001	0

Data are based on the analysis of 23 Armenian strains combined in the data set of 176 strains of *L*. *infantum* and the populations inferred by STRUCTURE. *F*_ST_ values are in the upper triangle, *p*-values in the lower triangle. Number of strains is given in parentheses.

**Fig 5:**
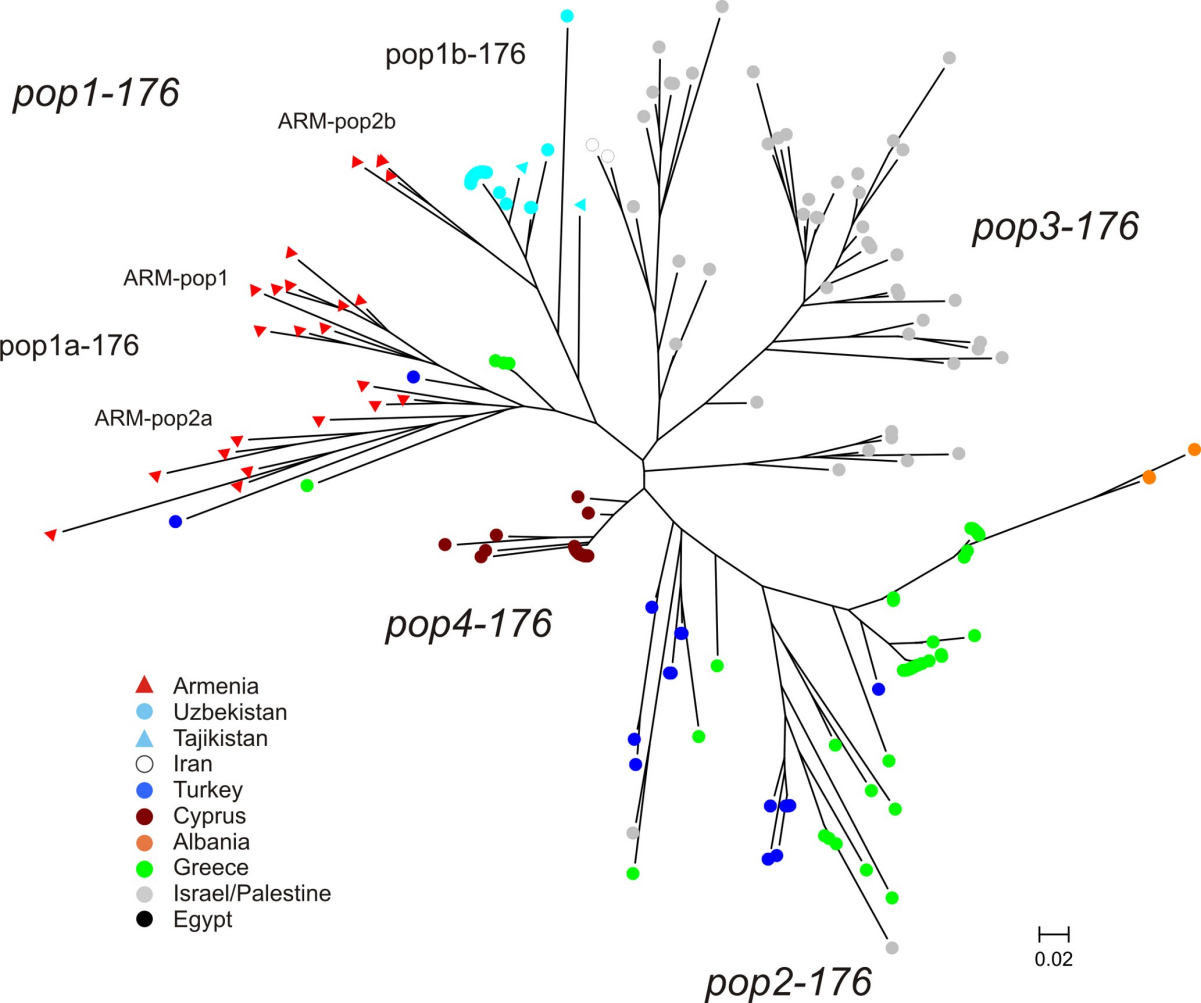
Neighbor joining tree inferred from the MLMT profiles of the subset of 176 *L*. *infantum* MON1 strains. The tree shows the position of *L*. *infantum* from Armenia in comparison to the most closely related subpopulations of the *L*. *infantum* populations of most endemic foci of the Old Word.

**Fig 5** shows the neighbor joining tree inferred from the MLMT data using the same dataset of 176 strains. In general the same clustering with very few differences could be observed as by the Bayesian inference, showing that the Armenian strains constitute a quite unique population together with the strains from Central Asia. This group splits into two subclusters, one of which contains all but four strains from Armenia (ARM-pop1 and ARM-pop2a), three strains from Greece and two from Turkey. This subcluster splits further into ARM-pop1 and ARM-pop2a. The other subcluster includes four strains from Armenia (ARM-pop2b), and all strains from Uzbekistan and Tajikistan. There is a difference in clustering of the Armenian strains with respect to ARM-pop2 depending on the size of the analyzed dataset, which was observed by STRUCTURE as well as by NJ-tree inference. Especially the position of the strains of ARM-pop2a is not stable. The number of strains and the genetic differences might not be sufficient for a clear differentiation of genetic groups, especially if the 23 Armenian strains were analyzed separately.

### Vector studies and diversity of the sand fly fauna in Armenia

**Fig 6:**
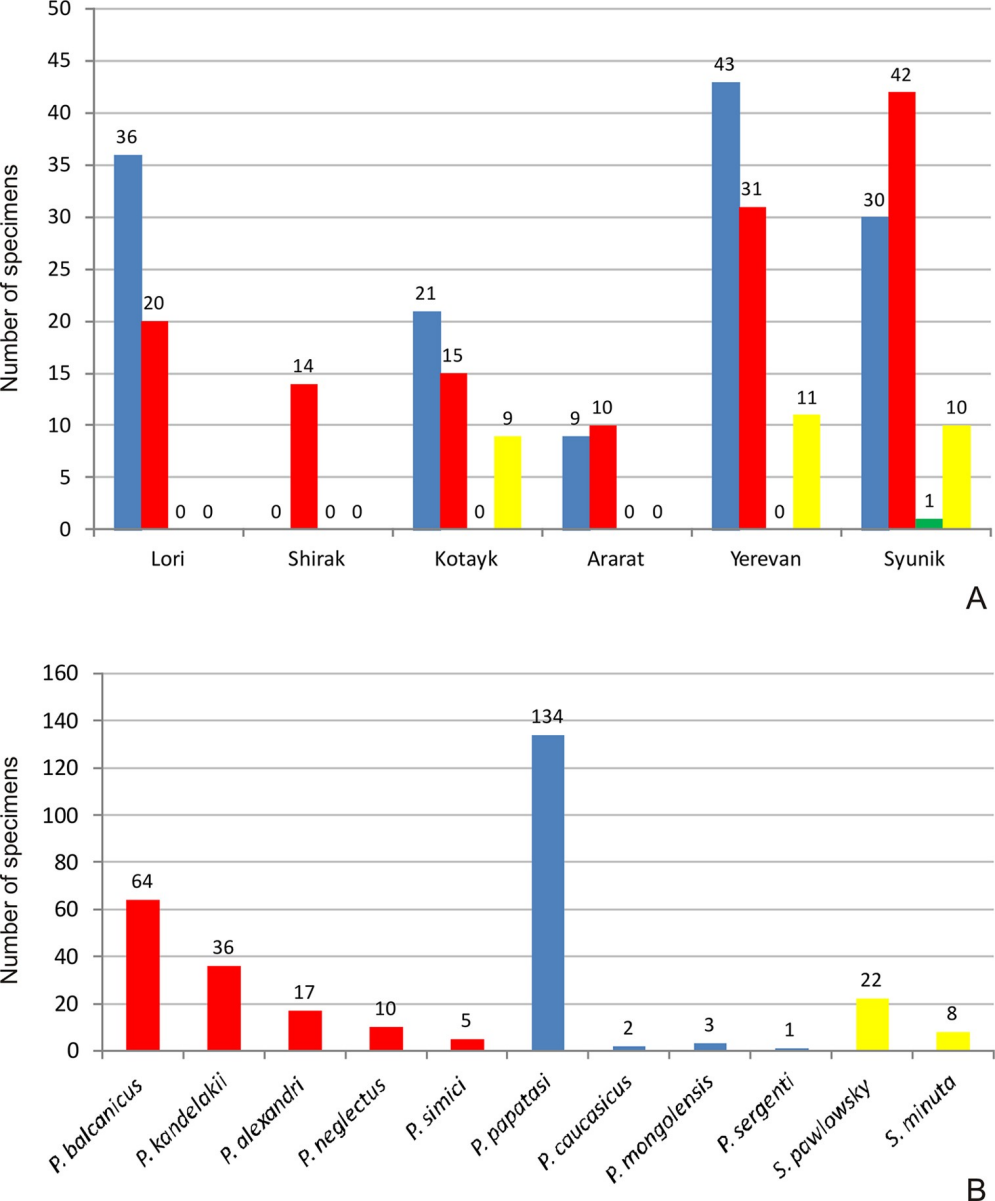
Diversity of sand fly species in Armenia observed during the vector surveys in active foci of visceral leishmaniasis. The surveys were conducted in six districts of Armenia in 2009–2015. The number of proven and potential vectors of *L*. *infantum* (red), *L*. *major* (blue), *L*. *tropica* (green) and *Sauroleishmania* (yellow) in the six districts is shown in (A). Eleven distinct sand fly species were collected. The numbers per species are shown in (B). Only two (*P*. *balcanicus* and *P*. *kandelakii*) are proven vectors of VL in the Southern Caucasus, three are potential vectors of *L*. *infantum* (red). *P*. *papatasi*, *P*. *caucasicus* (and *P*. *mongolensis* as morphotype of *P*. *caucasicus*) (blue) are proven vectors for zoonotic CL (*L*. *major*) in Central Asia, however *L*. *major* is absent in the Southern Caucasus. *P*. *sergenti* (green) is the potential vector for anthroponotic CL (*L*. *tropica)* in the Southern Caucasus. The two species of the subgenus *Sergentomyia* (yellow) are transmitting only *Sauroleishmania*.

Sand flies are occurring in Armenia from April until September/October. Vector surveys were carried out in active VL foci of Armenia located in the districts Yerevan, Syunik, Lori, Kotayk, Shirak and Ararat in 2009–2015. The results of the surveys including the amount of specimens per species, the location, altitude and year of collection are summarized in **S2 Table.** Eleven distinct species were identified (**Table 5 and Fig 6**). Out of these, only *P*. *balcanicus* and *P*. *kandelakii* are proven vectors of *L*. *infantum* in the Southern Caucasus, whereas *P*. *neglectus* and *P*. *simici* are considered as potential vectors in this region. *Phlebotomus papatasi* and *P*. *caucasicus* are proven vectors of *L*. *major* in Central Asia, but they obviously do not play a role in the Southern Caucasus since *L*. *major* is absent in Transcaucasia [21]. A single proven vector (*P*. *sergenti*) transmitting *L*. *tropica* was recorded.

Sand flies in general occurred in altitudes ranging from 637 to 2078 m. The proven and potential vectors of *L*. *infantum* were found in elevations from 637 to 2032 m. The most abundant species *P*. *papatasi* was trapped in elevations from 637 to 1746 m. All VL cases were recorded from 552 m to 2133 m, with the majority in altitudes of 1000–1500 m (76.4%), and 14.6% in 500–1000 m and 9% above 1500 m.

The diversity of sand fly species and the number of specimens collected in each surveyed district is shown in **Table 5.** The abundance per proven or potential vectors transmitting either *L*. *infantum*, *L*.*major*, *L*. *tropica* and *Sauroleishmania* in general (not restricted to Transcaucasia) is shown per district (A) or per species (B) in **Fig 6**. The most abundant species are the *L*. *major* vector *P*. *papatasi* (44%) and the proven *L*. *infantum* vectors *P*. *balcanicus* (21%) and *P*. *kandelakii* (12%). Species diversity was highest in Syunik, Kotayk, and Yerevan with seven, seven and six species, respectively. *Phlebotomus balcanicus* was found in all six districts. *Phlebotomus kandalakii* was not detected in Kotayk and Yerevan, instead *P*. *neglectus* and *P*. *alexandri* were found exclusively in these two districts. *Phlebotomus papatasi* was present in all districts except for Shirak. Neither *P*. *balcanicus* nor *P*. *kandelakii* were found only in two (Hrazdan river canyon and Sevaberd) of the 16 surveyed active foci. In the 14 remaining foci *P*. *balcanicus* was present in all but one (Meghri). *Phlebotomus kandelakii* was recognized only in six foci (Shnogh, Yeghegnut, Hartashen, Dvin, Goris, Meghri).

**Table 5:** Overview of sand fly species and the number of respective collected specimens in the surveyed districts.

Vector species	Subgenus	vector for ^1,2^	Lori	Kotayk	Shirak	Ararat	Yerevan	Syunik	Total
***P*. *(Ad*.*) balcanicus*** Theodor	*Adlerius*	*Leishmania infantum* ^*1*^	14	7	4	4	7	28	64
*P*. *(Ad*.*) simici* Nitzulescu	*Adlerius*	*Leishmania infantum*^*2*^					5		5
***P*. *(La*.*) kandelakii*** Shchurenkova	*Larroussius*	*Leishmania infantum*^*1*^	6		10	6		14	36
*P*. *(La*.*) neglectus* Tonnoir	*Larroussius*	*Leishmania infantum*^*2*^		3			7		10
*P*. *(Pa*.*) alexandri* Sinton	*Paraphlebotomus*	*Leishmania infantum*^*2*^		5			12		17
*P*. *(P*.*) papatasi* Scopoli	*Phlebotomus*	*Leishmania major*^*1*^	36	19		9	43	27	134
*P*. *(Pa*.*) caucasicus* Marzinowsky	*Paraphlebotomus*	*Leishmania major*^*1*^		2					2
*P*. *(Pa*.*) sergenti* Perfiliev	*Paraphlebotomus*	*Leishmania tropica*^*1*^						1	1
*P*. *(Pa*.*) mongolensis* Sinton^4^	*Paraphlebotomus*	*Leishmania major*^*2*^						3	3
*S*. *(S*.*) minuta* Rondani	*Sergentomyia*	*Sauroleishmania*^*3*^		5				3	8
*S*. *(S*.*) pawlowsky* Perfiliev	*Sergentomyia*	*Sauroleishmania*		4			11	7	22

^1^ Reported as proven vector for the respective *Leishmania* species in different countries; the two proven vectors for visceral leishmaniasis in Southern Caucasus are marked in bold letters;

^2^ Known as potential vector in different countries;

^3^ A first report about a naturally infected *S*. *minuta* with *L major* was published recently [67];

^4^ Considered as synonym of *P*. *caucasicus* [68,69], however the taxonomic position is not yet fully resolved.

### Spatiotemporal modeling and risk assessment of visceral leishmaniasis in Armenia

**Fig 7:**
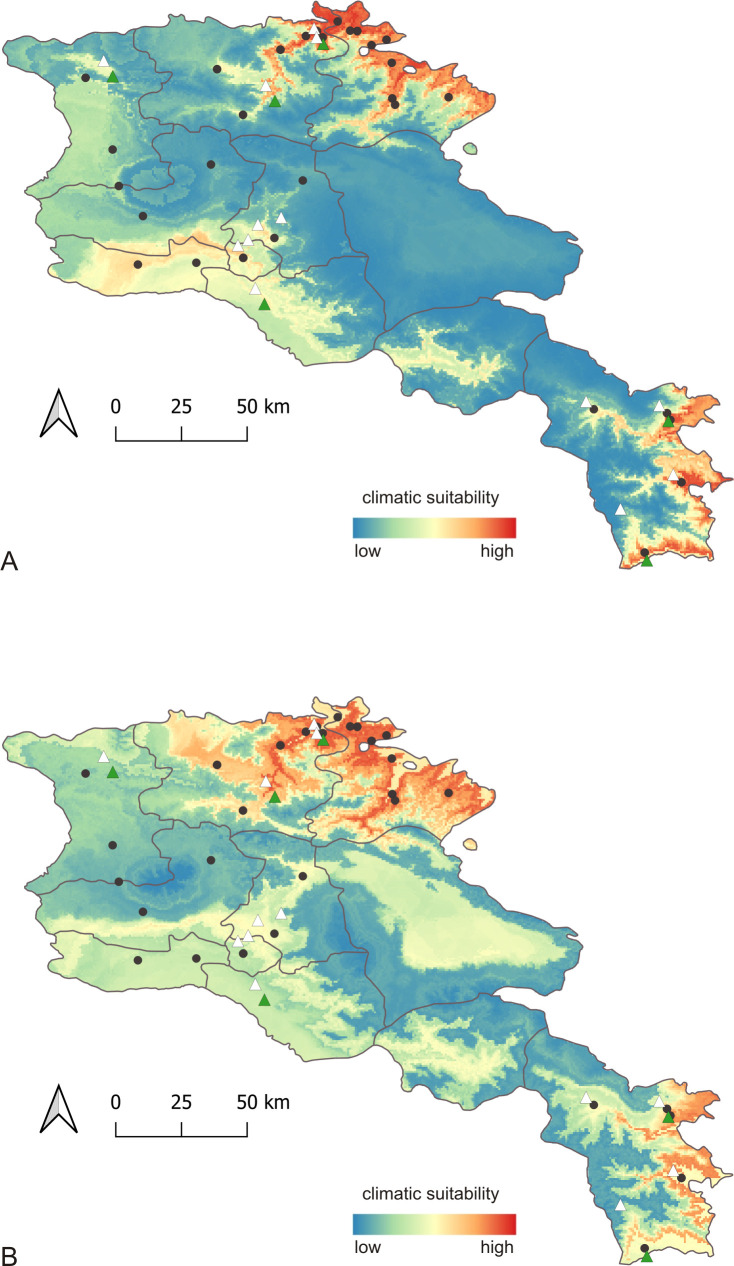
Inference of spatiotemporal projection using correlative ecological niche models for risk assessment of leishmaniasis. Modeled climatic suitability for the vectors transmitting *L*. *infantum—P*. *balcanicus* and *P*. *kandelakii—*and risk assessment of visceral leishmaniasis in Armenia (districts are shown). (A) current projection; (B) future projection for 2041–2060. Occurrence of vectors transmitting *L*. *infantum* is indicated by green (*P*. *kandelakii*) and white (*P*. *balcanicus*) triangles, cases of visceral leishmaniasis by black dots. The map was generated by the use of the shapefile of Armenia available from https://gadm.org/download_country_v3.html.

Spatiotemporal modeling based on climatic conditions revealed regions with different climatic suitability for disease transmission. The analysis included occurrence data of the two proven vectors of *L*. *infantum*, *P*. *kandelakii* and *P*. *balcanicus* from 2009–2015, as well as all 91 clinical cases of VL from the time period 2009–2016. The 91 VL cases were recorded in 30 different localities (23 cities, 7 villages) in eight districts. Proven vectors were collected in 13 localities (6 cities, 7 villages) in six districts **(S4 Table)**. **Fig 7** shows the present (A) and future (B) projections of the climatic suitability for VL transmission. The future projections were calculated for the time period 2041–60, using the CMIP5 future climate projections with GCM mpi-esm-lr and RCP 4.5 available from WorldClim 1.4. The regions with highest projected climatic suitability are located in the north of Armenia in the districts Lori and Tavush near the borders to Georgia (region Kwemo Kartli) and Azerbaijan (region Ganja-Qazakh). Another region of highest climatic suitability includes the eastern and southeastern parts of Syunik which are bordering to Southwestern Azerbaijan and the province East Azerbaijan in Northwest Iran. The third region at moderate to high climatic suitability is projected for the districts Yerevan, Armavir, Aragotsotn (southern parts) and Ararat (western and south western parts). Suitable areas in Ararat and Armavir border Turkey (province Iğdır in Eastern Anatolia). Regions with moderate projected suitability were predicted for parts of the districts Kotayk (southwest), the central part of Vayots Dzor and the North of Shirak which borders the Kars province in Turkey. Districts with no or very low suitability are Gegharkunik, eastern part of Kotayk, northern part of Aragotsotn and northwestern part of Syunik. Future projections for 2041–2060 predict a huge increase of regions with very high climatic suitability, especially almost all over the districts Tavush and Lori and a stable high suitability in southeastern and eastern Syunik. Yerevan, the south of Aragotsotn and Kotayk remain a region with high or moderate climatic suitability, depending on the impact of bioclimatic variables related to humidity. In general it should be expected that more regions, including those at higher altitudes, will be at risk for a further spread of VL because the climatic habitat suitability for sand flies will increase all over the country, e.g. in the west, the north and the southeast of the country.

## Discussion

### Genotypic diversity and position of Armenian *L*. *infantum* strains among the global *L*. *infantum* populations

The present study constitutes to our knowledge the very first genotyping study at strain level of the causative agent of VL in Armenia and in Transcaucasia. All clinical samples were identified as *L infantum* belonging to the worldwide predominating MON1 population. MON1 is one of the numerous zymodemes established for the genus *Leishmania* by Multilocus Enzyme Electrophoresis (MLEE, izoenzyme analysis), which for a long time was the gold standard for *Leishmania* typing [70]. MON1 strains represent more than 70% of all identified strains from VL patients and they are prevalent in infected dogs, which are considered as the main reservoir of *L*. *infantum* MON1 [71–73]. The identification of the Armenian strains as *L*. *infantum* MON1 is therefore of epidemiological importance in the context of further monitoring and control of VL in Armenia including also infected dogs. Differentiation and consequently epidemiological studies of MON1 strains were enabled quite recently with the development of standard sets of microsatellite markers [33,38]. The hypervariable microsatellite markers show the highest discriminative power apart from whole genome sequencing and genome-wide genotyping and can be used for clinical material without the need of cultivating the parasites. In previous studies we could demonstrate the existence of different geographically determined genotypic populations within the predominant zymodeme MON1 which has important epidemiological implications [37–40,42].

In the present study we could show that the Armenian strains, although originating from a rather small geographic area, are genetically divers. Among these samples three main groups could be identified, however without any correlation to the geographical origin in Armenia and the year of occurrence of the cases. This has to be verified by including more samples from the different foci in future studies. Historically, different natural foci of VL were known in Armenia [8,10]. The existence of natural barriers in this mountainous country and the presence of wild animal reservoirs that ensure the persistence of the parasites in different rural regions account for the maintenance of different subpopulations of the parasite. On the other side, the country is small and frequent traveling of people over the time can be assumed, which can lead to a distribution of different genotypes to different parts of the country. The introduction of new genotypes from active foci of neighboring countries is also feasible.

The comparison of the Armenian samples with worldwide occurring genotypes of *L*. *infantum* placed them by distance analysis as unique group among strains originating from Greece, Turkey, Cyprus, the Middle East, Central Asia, and a subpopulation comprising the majority of strains from Maghreb. These clusters were separated from the populations from Western Europe, the New World and China. Bayesian model-based analysis led to similar results with the difference that the majority of strains from Greece and Turkey were grouped at *K* = 3 together with the Western European strains (pop2-537) along with three strains from Armenia belonging to ARM-pop2. This revealed that the Greek, Turkish and few Armenian strains have an intermediate position between the two main MON1 population “Western Europe” (pop2-537) and “North Africa/Middle East/Cyprus/Central Asia” (pop3-537). However, the majority of Armenian strains were part of pop3-537, as also few strains from Greece and Turkey. The analysis of the intermediate group together with pop3-537 showed that there are two major genotypes in Armenia–the first one is occurring with lower frequency in Turkey and Greece, and the other one is present in Central Asia (Uzbekistan, Tajikistan). This is likely reflecting the geographic location of Armenia between Central Asia, the Middle East and Europe and the history of Armenia and Transcaucasia in general with frequent migration of populations, close trade relations among others along the Silk Road and the involved spread of infectious diseases. One of the main two trading routes between Imperial Russia and Persia led from Tiflis (now Tbilisi) through the former provinces Georgia and Armenia to Tebris (now the capital of the province East Azerbaijan in Iran). Later, during the existence of the USSR there were close relationships and traveling of people between the former Soviet republics in Central Asia and the Caucasus. The hypotheses about the historical mode of spread of VL has however to be verified by further studies including strains from the respective countries, regions and specific foci.

Apart from this work, very few molecular typing studies have been conducted on the causative agents of VL in Transcaucasia so far. In Georgia, sequencing of the ITS-region of 19 VL samples from dogs, humans and sand flies revealed genotypes specific for the *L*. *donovani* complex [74]. In Armenia, 23 strains were identified by RFLP analysis and sequencing of the ITS1 region as *L*. *infantum* [26]. Isoenzyme typing was performed in Transcaucasia only for single cases. In 1989 MLEE with a 12 enzyme system was performed for eight strains isolated from human VL cases from the Ordubad region in Azerbaijan [75]. It was shown that all these eight strains shared the same zymodeme among each other and with the included *L*. *infantum* reference strain MCAN/TN/1978/LEM78 which belongs to the MON1 zymodeme. The Ordubad region is bordering the district Syunik in Armenia, and in the present study strains from Syunik were identified as MON1 genotype. Another strain isolated from a CL outbreak caused by *L*. *infantum* in the Geokchai region (Azerbaijan) in 1984–86 represented a new zymodeme [76]. It is known that the majority of CL cases (~ 80%) are caused by other zymodemes than MON1. This is an indication that also other genotypes, which are more distantly related to the MON1 populations found in Armenia so far, are circulating in the Southern Caucasus region. Thus, the relationship between genotype and tropism should be further studied.

### Dynamics and possible reasons of re-emergence of visceral leishmaniasis

Another important question is related to the dynamics of re-emergence of VL in Armenia. In order to answer this question, the past and current epidemiological situation of the whole region of Southern Caucasus has to be considered. In the last decade the number of annual cases was 120–190 in Georgia, 60–110 in Azerbaijan, and 4–22 in Armenia, with a growing tendency [1,21,26,30]. Re-emergence was observed mainly in historically known old foci but also in several new ones. Old foci in Armenia were mainly in Yerevan and the Ararat plain, the cases were recorded in 16 of the 37 former districts in eight of a total of 22 Armenian cities and in 54 villages [10]. Since re-emergence of VL in Armenia in 1999 116 indigenous cases were recorded in eight of the eleven districts until 2016, with highest numbers of cases in Syunik (29%), Tavush (23%), Yerevan (22%) and Lori (19%) [26]. The number of cases in 2017–2019 remained stable with 17 cases in each year. It was also shown that the seroprevalence of VL in dogs was highest in Tavush (16.1%), followed by Syunik (9.3%) and Lori (6.5%) [28]. The modeling approach applied in the present study identified these regions as those with the highest climatic suitability for the vectors and hence the risk for VL transmission. Interestingly, all these regions are next to the border of countries and their specific regions, where a high incidence of VL was observed in the last decades. This indicates that the re-emergence of VL in Transcaucasia and its dynamics has to be studied in a broader context including all affected countries. It should be considered circulating *L*. *infantum* genotypes (from humans, dogs, wild animal reservoirs, and sand flies), the abundance of proven and potential vectors and wild animal reservoirs, seroprevalences in humans and dogs, biogeography, land use, topological specificities of the whole region and prognoses related to climate change. Unfortunately, no strains were available for MLMT in frame of this study from the neighboring countries Georgia and Azerbaijan, especially from the most adjacent provinces of these countries. Interestingly, the two strains from Iran typed in this study were not directly related to the Armenian strains, although they originated from the province Ardabil which is close to the district Syunik. There were also no strains from the neighboring active focus in Kars among the included Turkish strains.

A dramatic spread of the disease has been observed especially in Georgia since the re-emergence in 1990. Until 2010 1995 cases were registered, mainly from two foci–the Tbilisi region and the Kwemo-Kartli region (in the East) and Kutaisi (in the West) (Imereti region), with high seroprevalences in dogs as well as in humans [1,21,30]. In Armenia, the regions with the highest VL risk are located in the northern districts Tavush and Lori, next to the border to Georgia (region Kwemo Kartli), about 50 km from Tbilisi and Azerbaijan (region Ganja-Qazakh).

In Azerbaijan 347 cases were recorded from 1989 until 2009. Most of them occurred in northwestern Azerbaijan (Qazak, Tovuz) (next to Tavush in Armenia) and Shamkir. Other foci are in the central, eastern (Shamakhi) and southeastern (Jalilabad) parts of the country [1,21]. In Iran, the northwestern provinces East Azerbaijan and Ardabil are considered as the main endemic VL regions with also high seroprevalences in dogs [77–81]. The district Syunik in Armenia, with the second highest risk for VL, borders East Azerbaijan in Iran and southwestern Azerbaijan.

In Turkey, VL is endemic and one of the most affected foci is Kars (most northeastern part of Turkey in Eastern Anatolia) [82,83], that borders the district Shirak in Armenia, a region with moderate climatic suitability so far. Interestingly, Kars is a new focus and the only one in East Turkey. First reports of cases were from 1996–2002, mainly from the town Kagisman and several villages, which are situated at high altitude (>1300 m) and near the river Aras. Surveys on canine leishmaniasis revealed an incidence of 4–8% in the Kars and Iğdır provinces [83] which border the VL susceptible regions Ararat and Armavir in Armenia.

(Re)-emergence of VL occurred nearly at the same time period in Georgia, Armenia and Kars in Turkey in the 1990s. On the other side, in Iran VL always occurred sporadically, but since the 1970s the numbers increased considerably. A similar picture was observed in Azerbaijan, where VL never disappeared completely and a considerable increase of cases was recorded since 1984 [21].

Different reasons of re-emergence of VL in Transcaucasia could be considered, e.g. persisting rural foci and human intervention in rural regions (new settlements, agriculture, transport, etc.), the colonization of urban areas by wild animals as foxes, high density of pet and stray dogs in large cities, the changing climate, lack of awareness of the disease among the local population and doctors, and lack of modern diagnostic facilities and trained medical personal. After the collapse of the USSR, surveillance and control of vectors, infected dogs and patients was interrupted over a certain period and it had to be continuously re-organized. Also travelling, migration, and trading might play an important role, as regions with the highest risks for VL transmission in Armenia are located in the Debed (district Lori) and Aghstev (district Tavush) valleys with the main roads from the North into Armenia passing through the Lesser Caucasus. In Armenia VL foci were found predominantly in urban (2009–2016: 23 cities), but also in semi-urban and rural regions (2009–2016: 7 villages). The most active urban foci are the cities Yerevan (district Yerevan), Goris and Kapan (district Syunik), Akhtala and Alaverdi (district Lori), Idzhevan and Noyemberyan (district Tavush).

### Impact of topology and climate on vector occurrence and VL transmission: Projections based on climate change

Armenia has a variety of climates, such as dry continental climate (middle reaches of river Aras) with cold winters and hot summers, cold deserts in the lower parts of the Ararat plain or tundra on mountains peaks. According to the Köppen-Geiger climate classification, seven different climates (Cold desert climate, Bwk; Cold semi-arid climate, Bsk; Mediterranean-influenced warm-summer humid continental climate, Dsb; Mediterranean-influenced subarctic climate, Dsc; Subarctic climate, Dfc; Hot-summer humid continental climate, Dfa; Warm-summer humid continental climate, Dfb; Tundra climate, ET) are occurring in this small country of 29.800 km^2^ [84]. Regions identified as those with the highest VL susceptibility belong to the arid steppe (cold) (Bsk): (i) the Ararat plain, districts Armavir, Ararat, Yerevan, southern Aragotsotn and southwestern Kotayk; (ii) the regions next to the valleys of Debed and Aghstev in the districts Lori and Tavush as well as the foothills to the Kura-Aras depression; (iii) the most southern parts of the district Syunik around the valleys of the rivers Meghri, Vorotan, Voghchi and Aras; and partially to the category continental, no dry season, hot summer (Dfa) and warm summer (Dfb). Ninety percent of the country has an altitude > 1000 m, the mean altitude is 1800 m and only 3% lies below 650 m. Apart from the mountains (e.g. Lesser Caucasus in the North, Zangezur Mountains in the Southeast), there are lowlands (e.g Ararat, Shirak and Lori lowlands) and river valleys (e.g. of the rivers Aras, Hrazdan, Vorotan, Kassagh, Debed). There are strong seasonal temperature variations due to the mountainous character of the country and the moderating influences of the Mediterranean and Black Sea are blocked by mountain formations.

The currently active foci are spanning a wide range of altitudes from 552–2133 m, with most cases (76.4%) in the range 1000–1500 m. A similar observation was made also in the past, where the cases were found in altitudes from 620 until 1620 m, with 90% in the range 1000–1500 m [8,85]. However, there seems to be a slight trend of occurrence of VL in higher altitudes in the present. The sand flies *P*. *balcanicus* and *P*. *kandelakii* were found in the present study in an altitude range of 637–2078 m. According to previous studies in the 1960s, only these two species are typically found even at very high altitudes, in Armenia up to 1740 m, in Georgia 1240 m and in Azerbaijan 1700 m [8,15,16]. Old VL foci in Azerbaijan were recorded in 0–1050 m. In Georgia 64% were in 300–600 m, 33.5% in 600–1240 m [85]. The occurrence of cases at very high altitudes particularly in Armenia in comparison to the other countries can be explained by the specific topology of this country that enhances the continental character of the climate and leads to a vertical shift of natural zones into higher regions.

It was known from observations and studies in the 1970s that the area of VL circulation was very large in the Southern Caucasus. The very first detailed nosogeographical study was presented in 1970 and 1971 by Karapetyan and Bagdasyan [10,85], unfortunately available only in Russian. The authors identified seven climatic regions with active VL foci and presented a detailed map of the distribution of these climatic regions for Armenia, Azerbaijan and Georgia and mapped all VL foci. According to this study VL foci were located from the western shore of the Caspian Sea, the Kura depression, the Alazani valley and Tbilisi, the Kura-Aras lowland, the Aras valley, until the Ararat depression and plain. Further studies of the current re-emergence of VL in the Caucasus Region could be facilitated by having this map in mind.

The diversity of the sand fly fauna in Transcaucasia in general and in Armenia in particular, was very high in the past. More than 16 different species were recorded [8,15]. According to earlier studies, the most common species in Armenia is *P*. *balcanicus* (found in 14 of 16 former districts), followed by *P*. *papatasi* and *P*. *caucasicus* (in 12 districts), and *P*. *kandelak*i in 11 districts) [16]. The number of species as well as the quantity of sand flies decreased significantly in most foci after the extensive vector control measures in the 1960s, as it was shown in several studies in the 1970s and 1980s [13]. Recent entomological monitoring demonstrated an increase in vector populations of *P*. *balcanicus*, *P*. *kandelakii* and *P*. *papatasi*, especially in Yerevan, the Ararat valley, and the districts Syunik and Lori [44]. In the present study, at least one or both of the proven vectors of VL were found in 14 of the 16 surveyed foci. Nevertheless, the number of captured sand flies does not reflect the complete picture of the prevalence of the vectors. The low numbers do not correlate with the expected vector density needed to maintain VL transmission in the surveyed foci. Also not all of the most active foci have been investigated, e.g. in Tavush, Armavir and Aragotsotn, however it is known that these species are occurring in these districts. These limitations of the entomological survey were caused by limited resources. Further vector studies have to be performed in order to increase the robustness of a comparative analysis with the neighboring countries.

The observed diversity of vectors was high as eleven different species were recorded and all species were found even in very high altitudes. Vector diversity and composition in the neighboring foci in Turkey (Kars—ten species), Iran (East Azerbaijan, Ardabil—seven species) and Georgia (Kwemo Kartli, Tbilisi—five species) is similar as in Armenia [20,83,86–91].

The role of other species apart from *P*. *balcanicus* and *P*. *kandelakii* in the transmission of *L*. *infantum* in Armenia has to be clarified. Vector competences have not been proven for most of the species in Transcaucasia. Most incriminated vectors of *L*. *infantum* belong to the subgenus *Larroussius*, some to the closely related subgenus *Adlerius*. *Phlebotomus neglectus* and *P*. *simici* therefore could be considered as other potential candidates for the transmission of VL. *Phlebotomus neglectus* is a proven vector of VL in Greece and a suspected vector in the Balkans, Italy and Turkey [83,92–95]. *Phlebotomus alexandri* is a suspected vector in other countries, e.g. in Iran [96].

An extensive spread of *P*. *kandelakii* and *P*. *balcanicus* requires a temperature higher than 18°C over a longer period of time and an average humidity of 45–75% [85]. Both are anthropophilic and hygrophilous species, and especially *P*. *balcanicus* is known to be resistant to cold temperatures. There are different local climate conditions in Armenia due to significant altitudinal differences, which is one of the reasons for variation of the seasonal quantitative dynamics of sand flies in the different regions of the country.

It has been established that climate change, particularly warming, promotes the spread of many infectious and parasitic diseases such as a leishmaniasis or malaria [97–100]. Recent climate studies predicted a significant warming especially in Transcaucasia. In Armenia, the annual temperature increased between 1929 and 2011 by 1.03°C and the precipitation decreased by 6% in the monitored regions. A strong increase in temperatures was observed especially in the Ararat valley, the northeast (Tavush, Lori) and Meghri (Syunik) [98]. These regions also were identified in the present study as those with the highest risk of VL occurrence. In addition, Armenia is one of the five countries in Eastern Europe and Central Asia that will be most affected by hydrometeorological changes. The increase of temperature will spread to large territories in Armenia. As a result, more regions will have favorable climate and ecological (habitat) conditions which will further promote the spread of sand flies and increase the risk of VL transmission. This was also shown in the future projections in the present study, as the regions of moderate and high suitability for VL infections will spread over the entire country with hotspots in the current highly suitable regions. The risk for transmission of VL is increasing with the population density of the proven vectors. With increasing temperature the required development time of sand flies is reduced, and more than one generation can develop per year [101]. Also the development of *Leishmania* in the sand flies’ gut is accelerated by higher temperatures [102]. A study on the risk of renewed transmission of malaria in Armenia identified the same regions as hypermalariogenic as the present study for the risk for VL: Armavir, Kapan, Meghri, Yerevan, Artashat [98]. Also the altitudinal limit of the possible vertical spread of malaria is expected to shift to higher elevations.

The identified regions of high risk for VL in Armenia should be evaluated also in the general context of geography, topology, land use and climate in Transcaucasia. A first attempt has been made recently using spatial modeling by regression and fuzzy logic methods including apart from climatic and land cover variables also socio-ecological parameters as proximity to health care centers, proximity to nomadic villages, and population density [99]. Models for the identification of VL-susceptible areas were inferred based on data known from the most active VL focus in Iran (East Azerbaijan) and applied for the Southern Caucasus region. The authors identified the proximity to rivers, irrigated farming, and orchards as well as the population density as factors which increase the susceptibility for VL. Moreover, the models indicate that one of the most important sources of the persistence and transmission of VL throughout Transcaucasia could be the rural (nomadic) lifestyle. The inferred susceptibility map identified an area at the conjunction of the three countries Iran, Azerbaijan and Armenia, including one of the regions (district Syunik) identified in the present study as a highly suitable area.

## Conclusion and outlook

In the present study, the genetic diversity and population structure of the causative agent of visceral leishmaniasis in Armenia was addressed for the first time by using fast evolving microsatellite markers. The recent identification as *L*. *infantum* by PCR-RFLP was confirmed and all strains were assigned to the worldwide predominating MON1 population. The Armenian genotypes were compared with and assigned to specific geographic MON1 populations found in most endemic regions in the World. Based on the knowledge of the circulating genotypes of *L*. *infantum* in Armenia, further questions concerning the transmission cycles, the role of domestic dogs and wild animals as reservoirs as well as about the transmitting vector species can be addressed. In addition, it will allow the discrimination of indigenous from imported cases, the identification of the origin of infection and of epidemic outbreaks. Further population genetic studies should be performed with human and animal reservoir samples, especially from different active foci of the neighboring countries, to understand re-emergence, spread and epidemiology of the disease in Armenia and the entire Caucasus region and to enable epidemiological monitoring of VL. An important role for developing control strategies in the whole Southern Caucasus region will play further modeling based on a comprehensive collection of epidemiological and entomological data in this region. Perspectively, only coordinated surveillance and control measures between the healthcare systems of all countries of Southern Caucasus will prevent the persistence and re-emergence as well as the spread of this disease.

## Supporting information

S1 Table Overview of sand fly species reported from Armenia. (DOCX)

S2 Table Occurrence of distinct sand fly species in the respective active foci as collected during surveys from 2009–2015. (DOCX)

S3 Table List and geographic origin of the cases of visceral leishmaniasis included in the ecological niche modeling. (XLSX)

S4 Table Overview of the cities and villages in Armenia with occurrence of visceral leishmaniasis (2009–2016) and proven vectors (2009–2015) in Armenia. (DOCX)

S5 Table Results of Multilocus Microsatellite Typing (MLMT) for the 23 Armenian samples—fragment sizes for each of the 14 microsatellite markers. (XLSX)

S1 Fig Estimated population structure of 537 strains including *L*. *infantum* strains from endemic regions all over the Old World, the 23 Armenian samples and 12 representatives of *L*. *donovani* from the Middle East, Southeastern Europe and East Asia as inferred by Bayesian analysis of their MLMT profiles. In the barplots each strain is represented by a single vertical line divided into *K* colors, where *K* is the number of populations assumed. Each color represents one population. The length of the colors segment shows the strain’s estimated proportion of membership (Q) in that population. Strains are presented in the input order. According to Δ*K* the most probable number of populations is three (*K = 3*), in addition also *K =* 4 is shown.
(TIFF)

S2 Fig Estimated population structure of a subset of 176 *L*. *infantum* MON1 strains most closely related to the Armenian strains as inferred by Bayesian analysis of their MLMT profiles. In the barplots each strain is represented by a single vertical line divided into *K* colors, where *K* is the number of populations assumed. Each color represents one population. The length of the colors segment shows the strain’s estimated proportion of membership (Q) in that population. Strains are presented in the input order. (A) According to Δ*K* the most probable number of populations is four (*K =* 4), in addition also *K =* 3 is shown. (B) Structure of subpopulation 1 (pop1-176) comprising the strains from Armenia, Uzbekistan, Tajikistan and Turkey3. According to Δ*K* the most probable number of populations is two (*K =* 2).
(TIF)
