# Supplementary material for: Microsatellite based molecular epidemiology of Leishmania infantum from re-emerging foci of visceral leishmaniasis in Armenia and pilot risk assessment by ecological niche modeling
Source: PLoS Negl Trop Dis. 2021 Apr 19;15(4):e0009288. doi: 10.1371/journal.pntd.0009288 (PMC8055006; doi:10.1371/journal.pntd.0009288)
Supplement: S1 Table — (DOCX) [file pntd.0009288.s001.docx]

**S1 Table:** Overview of sand fly species reported from Armenia

| **Vector species** | **Subgenus** | **Potential or proven vector for ^1,2^** |
| --- | --- | --- |
| ***P. balcanicus*** Theodor | *Adlerius* | *Leishmania infantum ^1^* |
| *P. simici* Nitzulescu | *Adlerius* | *Leishmania infantum^2^* |
| *P. halepensis* Theodor | *Adlerius* | *Leishmania infantum^2^* |
| ***P. kandelakii*** Shchurenkova | *Larroussius* | *Leishmania infantum^1^* |
| *P. neglectus* Tonnoir | *Larroussius* | *Leishmania infantum^2^* |
| *P. perfiliewi* Parrot | *Larroussius* | *Leishmania infantum^2^* |
| *P. tobbi* Adler & Theodor | *Larroussius* | *Leishmania infantum ^1^* |
| *P. transcaucasicus* Perfilievi | *Larroussius* | *Leishmania infantum^2^* |
| *P. wenyoni* Adler & Theodor | *Larroussius* | *Leishmania infantum* |
| *P. syriacus* Adler & Theodor | *Larroussius* | *Leishmania infantum* |
| *P. papatasi* Scopoli | *Phlebotomus* | *Leishmania major^1^* |
| *P. alexandri* Sinton | *Paraphlebotomus* | *Leishmania infantum^2^* |
| *P. caucasicus* Marzinowsky  [syn. *P. grimmi* Portschinsky] | *Paraphlebotomus* | *Leishmania major^1^* |
| *P. sergenti* Perfiliev | *Paraphlebotomus* | *Leishmania tropica^1^* |
| *P. mongolensis* Sinton^4^ | *Paraphlebotomus* | *Leishmania major^2^* |
| *P. jacusieli* Theodor | *Paraphlebotomus* | *n.d.* |
| *S. minuta* Rondani | *Sergentomyia* | *Sauroleishmania^3^* |
| *S. pawlowsky* Perfiliev | *Sergentomyia* | *Sauroleishmania* |

^1^ Reported as proven vector for the respective *Leishmania* species in different countries; the two proven vectors for visceral leishmaniasis in Southern Caucasus are marked in bold letters; ^2^ Known as potential vector in different countries; ^3^ A first report about a naturally infected *S. minuta* with *L. major* was published recently [1]; ^4^ Considered as synonym of *P. caucasicus* [2-3], however the taxonomic position is not yet fully resolved.

References

1. Jaouadi K, Ghawar W, Salem S, Gharbi M, Bettaieb J, Yazidi R, et al. First report of naturally infected *Sergentomyia minut*a with *Leishmania major* in Tunisia. Parasit Vectors. 2015;8:6.
2. Parvizi P, Taherkhani H, Ready PD. *Phlebotomus caucasicus* and *Phlebotomus mongolensis* (Diptera:Psychodidae): indistinguishable by the mitochondrial cytochrome b gene in Iran. Bull Entomol Res. 2010;100(4):415-20.
3. Moin-Vaziri V, Depaquit J, Yaghoobi-Ershadi MR, Oshaghi MA, Derakhshandeh-Peykar P, Ferte H, et al. Geographical variation in populations of *Phlebotomus* (*Paraphlebotomus*) *caucasicus* (Diptera: Psychodidae) in Iran. Bulletin de la Societe de pathologie exotique (1990). 2007;100(4):291-5.
