# Supplementary material for: Microsatellite based molecular epidemiology of Leishmania infantum from re-emerging foci of visceral leishmaniasis in Armenia and pilot risk assessment by ecological niche modeling
Source: PLoS Negl Trop Dis. 2021 Apr 19;15(4):e0009288. doi: 10.1371/journal.pntd.0009288 (PMC8055006; doi:10.1371/journal.pntd.0009288)
Supplement: S2 Table — (DOCX) [file pntd.0009288.s002.docx]

**S2 Table:** Occurrence of distinct sand fly species in the respective active foci as collected during surveys from 2009-2015

| **District** | **City/Village** | **Latitude** | **Longitude** | **Altitude** | **Vector species** | **Number** | **Year** |
| --- | --- | --- | --- | --- | --- | --- | --- |
| Lori | Shnogh (v) | 41.150409 | 44.840012 | 637 m | *P. kandelakii* | 4 | 2013 |
|  |  |  |  |  | *P. balcanicus* | 6 | 2013 |
|  |  |  |  |  | *P. papatasi* | 14 | 2013 |
|  | Teghut (v) | 41.110844 | 44.850826 | 745 m | *P. balcanicus* | 3 | 2013 |
|  |  |  |  |  | *P. papatasi* | 8 | 2013 |
|  | Yeghegnut (v) | 40.901938 | 44.629675 | 1068 m | *P. kandelakii* | 2 | 2014 |
|  |  |  |  |  | *P. balcanicus* | 5 | 2014 |
|  |  |  |  |  | *P. papatasi* | 14 | 2014 |
| Shirak | Hartashen (v) | 41.009324 | 43.92334 | 2032 m | *P. kandelakii* | 10 | 2010 |
|  |  |  |  |  | *P. balcanicus* | 4 | 2010 |
| Kotyak | Sevaberd (v) | 40.2775 | 44.798333 | 2078 m | *P. caucasicus* | 2 | 2009 |
|  |  |  |  |  | *S. minuta* | 5 | 2009 |
|  |  |  |  |  | *S. pawlowsky* | 4 | 2009 |
|  | Kaputan (v) | 40.327011 | 44.696503 | 1746 m | *P. balcanicus* | 2 | 2009 |
|  |  |  |  |  | *P. papatasi* | 6 | 2009 |
|  |  |  |  |  | *P. alexandri* | 5 | 2009 |
|  | Arzni (v) | 40.296484 | 44.595219 | 1344 m | *P. papatasi* | 13 | 2015 |
|  |  |  |  |  | *P. balcanicus* | 5 | 2015 |
|  |  |  |  |  | *P. neglectus* | 3 | 2015 |
| Ararat | Dvin (v) | 40.017944 | 44.584064 | 924 m | *P. kandelakii* | 6 | 2010 |
|  |  |  |  |  | *P. balcanicus* | 4 | 2010 |
|  |  |  |  |  | *P. papatasi* | 9 | 2010 |
| Yerevan | Yerevan (c)  (Aygedzor) | 40.205563 | 44.508221 | 1159 m | *P. balcanicus* | 5 | 2012 |
|  |  |  |  |  | *P. papatasi* | 11 | 2012 |
|  |  |  |  |  | *P. neglectus* | 1 | 2012 |
|  |  |  |  |  | *P. alexandri* | 4 | 2012 |
|  | Yerevan (c) (Noragyugh) | 40.167349 | 44.492785 | 985 m | *P. simici* | 5 | 2012 |
|  |  |  |  |  | *S. pawlowsky* | 11 | 2012 |
|  |  |  |  |  | *P. alexandri* | 8 | 2012 |
|  |  |  |  |  | *P. papatasi* | 18 | 2012 |
|  |  |  |  |  | *P. neglectus* | 6 | 2012 |
|  |  |  |  |  | *P. papatasi* | 8 | 2015 |
|  | Yerevan (c) (Kanaker) | 40.229803 | 44.552786 | 1318 m | *P. papatasi* | 6 | 2014 |
|  |  |  |  |  | *P. balcanicus* | 2 | 2014 |
| Syunik | Goris (c) | 39.508494 | 46.343904 | 1332 m | *P. balcanicus* | 12 | 2010 |
|  |  |  |  |  | *P. kandelakii* | 7 | 2010 |
|  |  |  |  |  | *S. minuta* | 3 | 2010 |
|  | Meghri (c) | 38.90191 | 46.25004 | 673 m | *P. kandelakii* | 7 | 2011 |
|  |  |  |  |  | *P. papatasi* | 8 | 2011 |
|  | Lichk (v) | 39.057898 | 46.17485 | 1821 m | *P. balcanicus* | 9 | 2011 |
|  |  |  |  |  | *P. sergenti* | 1 | 2011 |
|  |  |  |  |  | *S. pawlowsky* | 4 | 2011 |
|  | Kapan (c) | 39.207552 | 46.404824 | 790 m | *P. balcanicus* | 5 | 2014 |
|  |  |  |  |  | *P. papatasi* | 11 | 2014 |
|  |  |  |  |  | *S. pawlowsky* | 3 | 2014 |
|  | Sisian (c) | 39.525595 | 46.025059 | 1600 m | *P. balcanicus* | 2 | 2015 |
|  |  |  |  |  | *P. papatasi* | 8 | 2015 |
|  |  |  |  |  | *P. mongolensis** | 3 | 2015 |

* *P. mongolensis* and *P. caucasicus* are distinguishable morphologically, however indistinguishable by the *cyt*b gene, synonymy is hence not yet clarified [1]. c – city, v -village

Reference

1. Parvizi P, Taherkhani H, Ready PD. *Phlebotomus caucasicus* and *Phlebotomus mongolensis* (Diptera:Psychodidae): indistinguishable by the mitochondrial cytochrome b gene in Iran. Bull Entomol Res. 2010;100(4):415-20.
