# Supplementary material for: Microsatellite based molecular epidemiology of Leishmania infantum from re-emerging foci of visceral leishmaniasis in Armenia and pilot risk assessment by ecological niche modeling
Source: PLoS Negl Trop Dis. 2021 Apr 19;15(4):e0009288. doi: 10.1371/journal.pntd.0009288 (PMC8055006; doi:10.1371/journal.pntd.0009288)
Supplement: S4 Table — (DOCX) [file pntd.0009288.s004.docx]

**S4 Table:** Overview of the cities and villages in Armenia with occurrence of visceral leishmaniasis (2009-2016) and proven vectors (2009-2015) in Armenia

| **District** | **City/Village** | **city** | **village** | **No. of VL cases** | ***P. balcanicus*** | ***P. kandelakii*** |
| --- | --- | --- | --- | --- | --- | --- |
| Aragatsotn | Aparan | x |  | 1 |  |  |
|  | Garnahovit |  | x | 1 |  |  |
|  | Otevan |  | x | 1 |  |  |
| Ararat | Dvin |  | x |  | 4 | 6 |
| Armavir | Armavir | x |  | 1 |  |  |
|  | Echmiadzin | x |  | 1 |  |  |
| Kotayk | Abovyan | x |  | 1 |  |  |
|  | Arzni |  | x |  | 5 | 0 |
|  | Kaputan |  | x |  | 2 | 0 |
|  | Hrazdan | x |  | 1 |  |  |
| Lori | Akhtala | x |  | 3 |  |  |
|  | Alaverdi | x |  | 6 |  |  |
|  | Mets Ayrum |  | x | 1 |  |  |
|  | Shnogh | x |  | 1 | 6 | 4 |
|  | Stepanavan | x |  | 3 |  |  |
|  | Teghut |  | x |  | 3 | 0 |
|  | Vanadzor | x |  | 2 |  |  |
|  | Yeghegnut |  | x |  | 5 | 2 |
| Shirak | Hartashen |  | x |  | 4 | 10 |
|  | Hovtun |  | x | 2 |  |  |
|  | Nor Kyank |  | x | 1 |  |  |
| Syunik | Goris | x |  | 5 | 12 | 7 |
|  | Kapan | x |  | 19 | 5 | 0 |
|  | Karahunj |  | x | 1 |  |  |
|  | Lichk |  | x |  | 9 | 0 |
|  | Meghri | x |  | 3 | 0 | 7 |
|  | Sisian | x |  | 1 | 2 | 0 |
| Tavush | Noyemberyan | x |  | 5 |  |  |
|  | Sarigyugh | x |  | 1 |  |  |
|  | Baganis | x |  | 2 |  |  |
|  | Berd | x |  | 1 |  |  |
|  | Gandzakar | x |  | 1 |  |  |
|  | Haghtanak | x |  | 1 |  |  |
|  | Ijevan | x |  | 2 |  |  |
|  | Koghb |  | x | 1 |  |  |
|  | Koti | x |  | 3 |  |  |
| Yerevan | Yerevan | x |  | 19 | 5 | 2 |
| **total** | **39** | **23** | **14** | **91** | **64** | **36** |

Cities or villages without entries in the columns for *P. kandelakii* and *P. balcanicus* were not included in the vector survey.
