# Supplementary figures and images for: Microsatellite based molecular epidemiology of Leishmania infantum from re-emerging foci of visceral leishmaniasis in Armenia and pilot risk assessment by ecological niche modeling
Source: PLoS Negl Trop Dis. 2021 Apr 19;15(4):e0009288. doi: 10.1371/journal.pntd.0009288 (PMC8055006; doi:10.1371/journal.pntd.0009288)

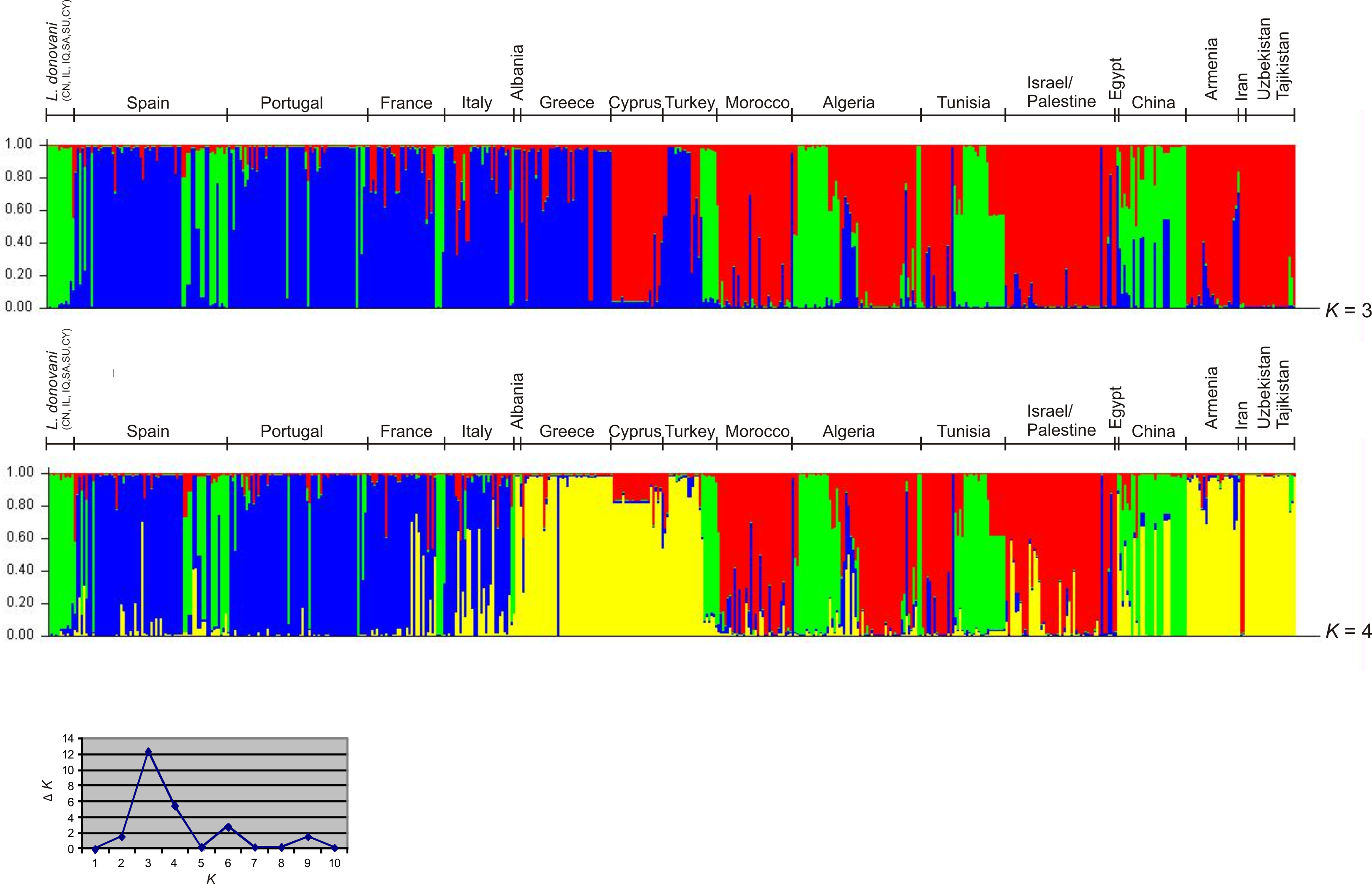

Supplement: S1 Fig — In the barplots each strain is represented by a single vertical line divided into K colors, where K is the number of populations assumed. Each color represents one population. The length of the colors segment shows the strain’s estimated proportion of membership (Q) in that population. Strains are presented in the input order. According to ΔK the most probable number of populations is three (K = 3), in addition also K = 4 is shown. (TIFF) [file pntd.0009288.s006.tiff]

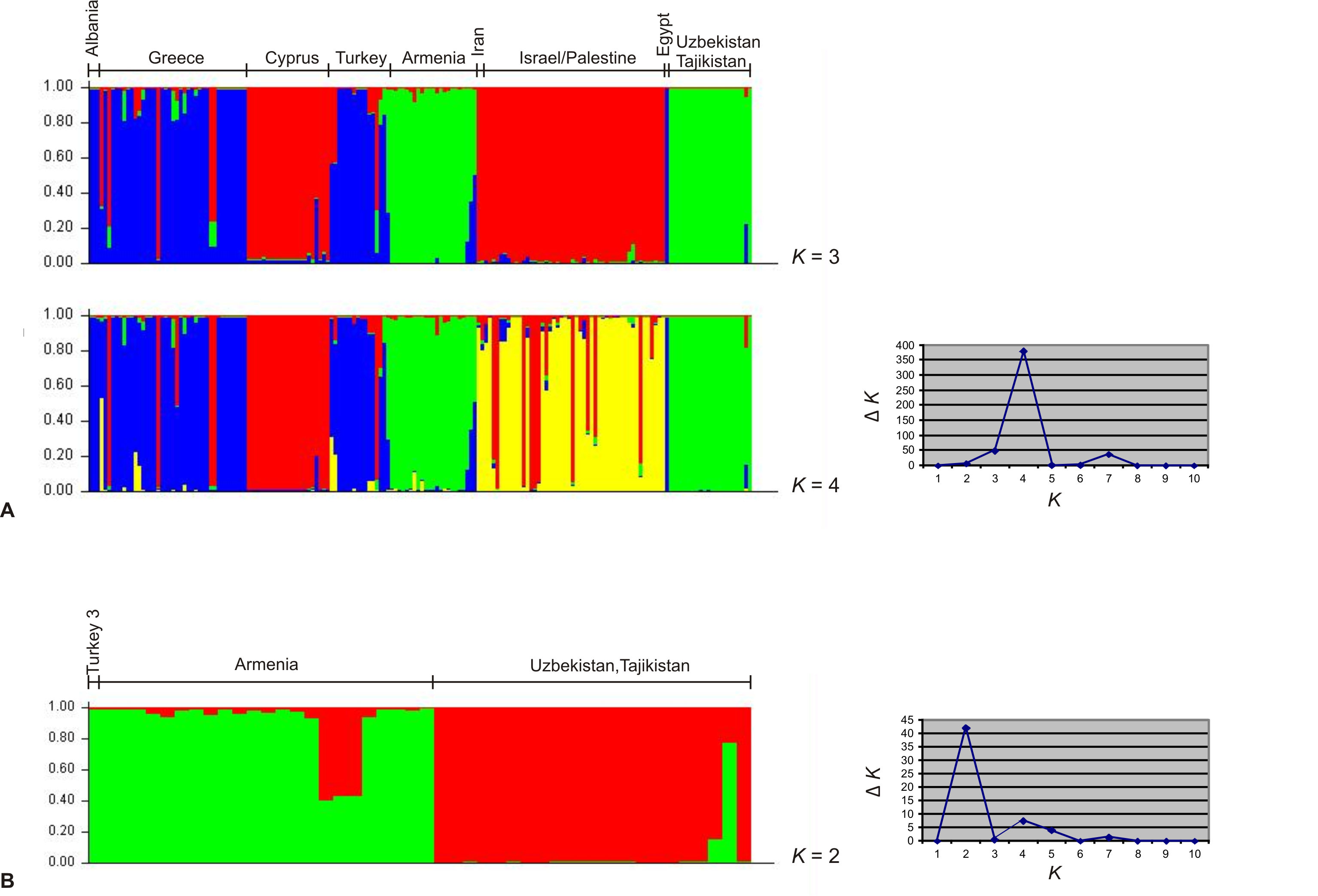

Supplement: S2 Fig — In the barplots each strain is represented by a single vertical line divided into K colors, where K is the number of populations assumed. Each color represents one population. The length of the colors segment shows the strain’s estimated proportion of membership (Q) in that population. Strains are presented in the input order. (A) According to ΔK the most probable number of populations is four (K = 4), in addition also K = 3 is shown. (B) Structure of subpopulation 1 (pop1-176) comprising the strains from Armenia, Uzbekistan, Tajikistan and Turkey3. According to ΔK the most probable number of populations is two (K = 2). (TIF) [file pntd.0009288.s007.tif]
